# Suppression of Adipogenesis by Pathogenic Seipin Mutant Is Associated with Inflammatory Response

**DOI:** 10.1371/journal.pone.0057874

**Published:** 2013-03-08

**Authors:** Wenjie Qiu, Kenneth Wee, Kosuke Takeda, Xuemei Lim, Shigeki Sugii, George K. Radda, Weiping Han

**Affiliations:** 1 Laboratory of Metabolic Medicine, Singapore Bioimaging Consortium, Agency for Science, Technology and Research, Singapore, Republic of Singapore; 2 Cardiovascular and Metabolic Disorders Program, Duke-National University of Singapore Graduate Medical School, Singapore, Republic of Singapore; 3 Department of Biochemistry, Yong Loo Lin School of Medicine, National University of Singapore, Singapore, Republic of Singapore; 4 Metabolism in Human Diseases, Institute of Molecular and Cell Biology, Agency for Science, Technology and Research, Singapore, Republic of Singapore; University of Bari, Italy

## Abstract

**Background:**

While pathogenic mutations in *BSCL2/Seipin* cause congenital generalized lipodystrophy, the underlying mechanism is largely unknown. In this study, we investigated whether and how the pathogenic missense A212P mutation of Seipin (Seipin-A212P) inhibits adipogenesis.

**Methodology/Results:**

We analyzed gene expression and lipid accumulation in stable 3T3-L1 cell lines expressing wild type (3T3-WT), non-lipodystrophic mutants N88S (3T3-N88S) and S90L (3T3-S90L), or lipodystrophic mutant A212P Seipin (3T3-A212P). When treated with adipogenic cocktail, 3T3-WT, 3T3-N88S and 3T3-S90L cells exhibited proper differentiation into mature adipocytes, indistinguishable from control 3T3-L1 cells. In contrast, adipogenesis was significantly impaired in 3T3-A212P cells. The defective adipogenesis in 3T3-A212P cells could be partially rescued by either PPARγ agonist or PPARγ overexpression. Gene expression profiling by microarray revealed that inhibition of adipogenesis was associated with activation of inflammatory genes including IL-6 and iNOS. We further demonstrated that Seipin-A212P expression at pre-differentiation stages significantly activated inflammatory responses by using an inducible expression system. The inflammation-associated inhibition of adipogenesis could be rescued by treatment with anti-inflammatory agents.

**Conclusions:**

These results suggest that pathogenic Seipin-A212P inhibits adipogenesis and the inhibition is associated with activation of inflammatory pathways at pre-differentiation stages. Use of anti-inflammatory drugs may be a potential strategy for the treatment of lipodystrophy.

## Introduction

Congenital generalized lipodystrophy (CGL), also known as Berardinelli-Seip congenital lipodystrophy (BSCL), is a rare autosomal recessive disease characterized by the near total absence of adipose tissue from birth or early infancy [1]. Affected patients often develop metabolic syndrome similar to those suffering from obesity-associated metabolic diseases [1].

Studies to date have mapped CGL to four different chromosomal loci, namely *BSCL1* (9q34), *BSCL2* (11q13), *caveolin-1* (7q31) and *PTRF/Cavin* (17q21). *BSCL1* encodes for the 1-acylglycerol-3-phosphate-O-acyltransferase 2 (AGPAT2) protein, a key enzyme in the synthesis of triacylglycerol (TAG) and phospholipids from glycerol-3-phosphate [2], [3], [4]. The *BSCL2* gene encodes for the protein Seipin, a molecule hypothesized to be involved in the regulation of adipogenesis and the formation of lipid droplet (LD) [5], [6], [7]. A more recently established CGL3 related protein caveolin-1 (Cav-1) was identified as an essential component of caveolae [8], and a fatty-acid binding protein with a potential role in lipid transport, lipolysis and LD formation [9]. Another protein essential for caveolae biogenesis, PTRF-Cavin, was found responsible for a novel lipodystrophic subtype, CGL4 [10], [11].

Although CGL2 patients have a more severe phenotype than the other CGL patients, the molecular function of its encoded protein, Seipin, is unknown. YLR404W/Fld1p, a Seipin functional ortholog in budding yeast, was suggested to be involved in LD assembly and/or maintenance through the regulation of phospholipid synthesis [6], [12]. In mammalian pre-adipocyte models, adipogenesis was impaired in the absence of the murine Seipin ortholog, and the impairment was associated with down-regulation of adipogenic transcription factors and lack of lipid accumulation [7], [13]. These results suggest that Seipin or its functional orthologs may have diverse functions in specific cell types, a notion that is supported by a recent genetic study [14].

So far, at least 30 Seipin mutations have been discovered to be associated with lipodystrophy. Except for certain missense mutations, such as A212P, most of the mutations contain nonsense, frame-shift or aberrant splicing mutations that produce truncated, non-functional proteins [15]. Two missense mutations, N88S and S90L, which are known to cause motor neuropathy in a autosomal dominant manner [16], have not been reported to be associated with adipogenic defects or lipodystrophy. While Seipin is required for PPARγ activation, it remains unclear how Seipin regulates adipogenesis, and whether and how various Seipin mutants cause lipodystrophy. Here, we demonstrated that Seipin-A212P inhibited adipogenesis by down-regulation of PPARγ expression in 3T3-L1 cells. This defect could be partially rescued through treatment with a PPARγ agonist or PPARγ overexpression. Furthermore, we discovered that the inhibition in adipogenesis was associated with an activated inflammatory response, and Seipin-A212P expression at pre-differentiation stages significantly activated inflammatory responses. Together these results suggest that the missense A212P Seipin mutant inhibits adipogenesis and the inhibition is associated with inflammatory responses.

## Results

### Seipin-A212P Inhibits Adipogenesis in 3T3-L1 Pre-adipocytes

**Figure 1:**
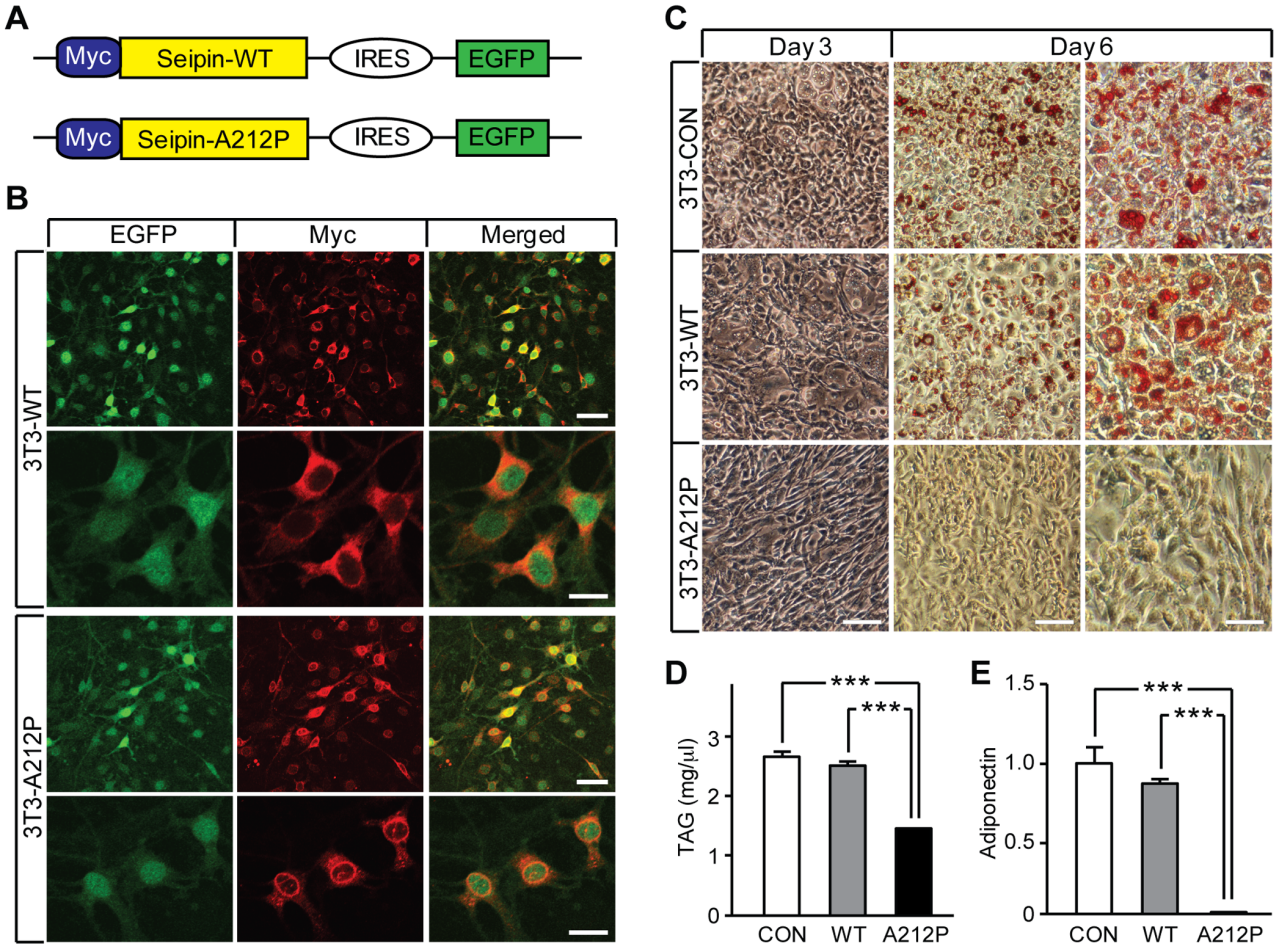
Seipin-A212P inhibits adipogenesis in 3T3-L1 cells. (A) A schematic diagram of the constructs used in generating the two stable 3T3-L1 cell lines expressing wild type Seipin (3T3-WT) or A212P mutant (3T3-A212P). Both Seipin-WT and Seipin-A212P were tagged with Myc at the N-terminus and co-expressed with EGFP by linking the two coding sequences with IRES. (B) Confocal images showing 3T3-WT and 3T3-A212P stable cell lines expressing EGFP and Seipin (Myc) proteins. Scale bars = 50, 25, 50 and 25 µm, respectively. (C) Cells expressing empty vector (3T3-CON), 3T3-WT or 3T3-A212P cells were treated with the standard adipogenic cocktail, and lipid accumulation was assessed by Oil Red O staining at day 6. Phase contrast microscopy showing different morphological changes in 3 cell lines at day 3. Scale bars = 100, 100, and 50 µm, respectively, and apply to each column of images. (D) Lipid was extracted at day 6, and total TAG contents of 3T3-CON, 3T3-WT and 3T3-A212P cells were measured. (E) Relative level of adiponectin expression in 3T3-CON, 3T3-WT and 3T3-A212P cells were analyzed by real-time qPCR. Data are presented as mean ± SEM. N = 3 independent experiments, each measured in triplicates; ***p<0.001.

To understand the function of Seipin in adipocyte development and how Seipin-A212P affects adipocyte differentiation, we established stable 3T3-L1 cell lines expressing Seipin wild type (3T3-WT) or Seipin-A212P (3T3-A212P) by lentiviral transduction and FACS sorting. Seipin-WT and Seipin-A212P were tagged with Myc and followed by an internal ribosome entry site (IRES) and EGFP coding region (Figure 1A). As a control, we also generated a stable 3T3-L1 cell line carrying only the IRES and EGFP sequences (3T3-CON). GFP and Myc signals were readily detectable in stable 3T3-L1 cells, indicative of successful transduction of Seipin-WT or Seipin-A212P protein (Figure 1B). In stable 3T3-L1 cells, Seipin-WT localized in the cytoplasm, while Seipin-A212P was enriched around the nucleus (Figure 1B), consistent with a previous study [7].

We then treated these cell lines with standard adipogenic cocktail containing dexamethasone, IBMX and insulin (DMI) to induce adipocyte differentiation. On the third day post treatment (dpt), 3T3-A212P cells maintained fibroblast-like pre-adipocyte morphology, while 3T3-WT and 3T3-CON cells underwent normal differentiation, as evidenced by lipid accumulation and LD formation (Figure 1C). By 6 dpt, the differences became much more significant. 3T3-A212P cells exhibited a clear pre-adipocyte morphology, significant defect in lipid accumulation and LD formation, and an almost 50% reduction in total triacylglycerol (TAG) levels (Figure 1C and D). In contrast, 3T3-WT cells showed indistinguishable time course of adipocyte differentiation as 3T3-CON cells, and the total TAG content was the same as that in 3T3-CON cells. Adiponectin expression was decreased in 3T3-A212P cells when compared with 3T3-WT or 3T3-CON cells (Figure 1E). There was no difference in cell viability, indicating that these defects were not due to increased apoptosis (Figure S1). As the A212P mutation leads to Seipin misfolding, we examined whether the adipogenic defects were due to protein misfolding-associated ER stress by testing two other Seipin misfolding mutants Seipin-N88S and Seipin-S90L, and an unrelated protein α-synuclein. Expression of Seipin-N88S or Seipin-S90L mutant protein did not lead to defective adipogenesis. Similarly, expression of α-synuclein-WT (wild-type) or its misfolding mutant α-synuclein-A30P in 3T3-L1 cells did not inhibit adipogenesis (Figure S2). These results suggest that Seipin-A212P specifically inhibits adipocyte differentiation, lipid synthesis and/or storage via a mechanism more complex than protein misfolding-associated ER stress.

### Expression of PPARγ and Adipogenic Genes is Inhibited by Seipin-A212P

**Figure 2:**
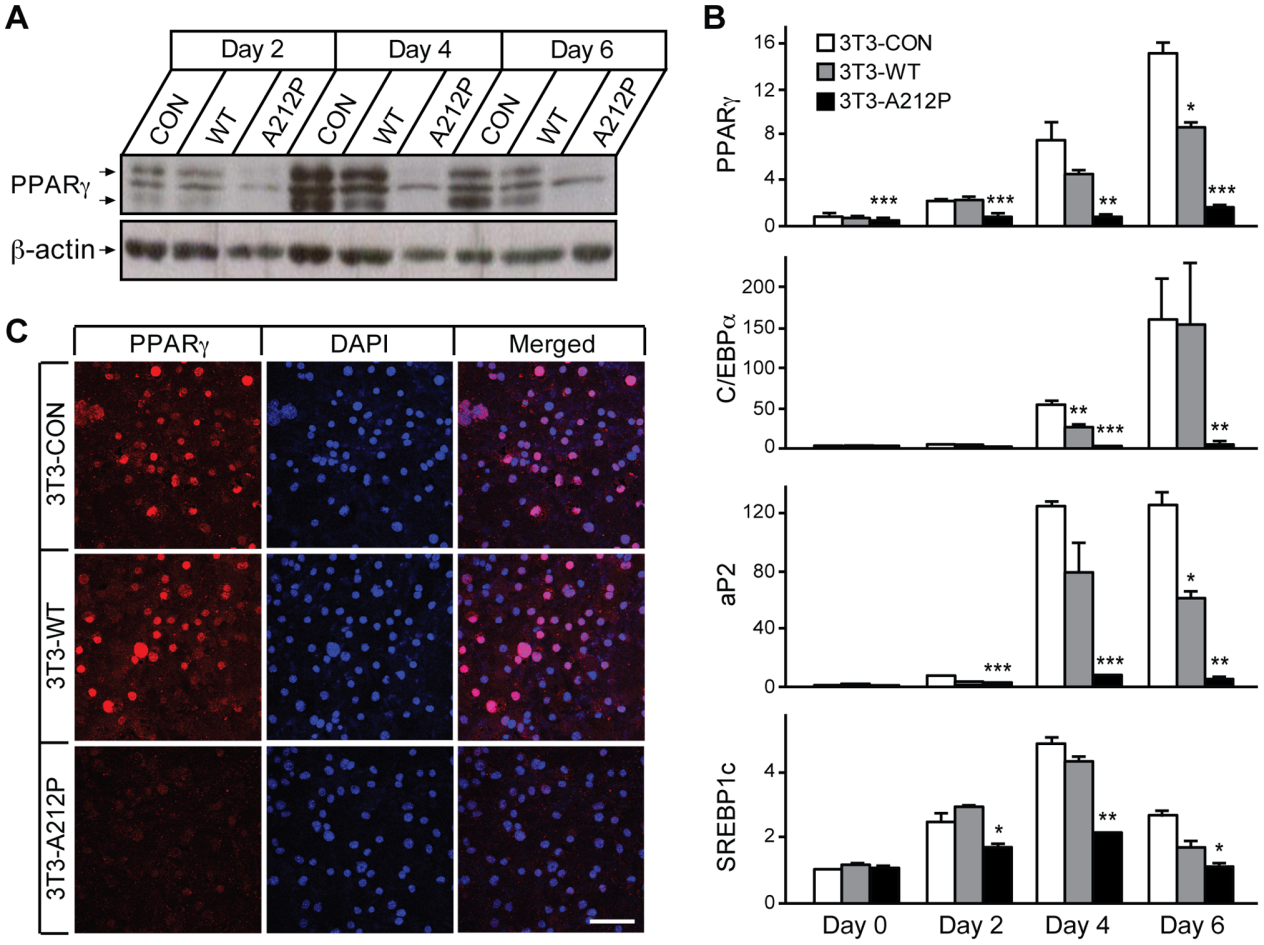
PPARγ expression is down-regulated in 3T3-A212P cells. (A) Western blot using an anti-PPARγ antibody showing PPARγ protein level in 3T3-CON, 3T3-WT and 3T3-A212P cells at the indicated time points during adipocyte differentiation. β-actin was used as a loading control. (B) Expression levels of PPARγ, C/EBPα, aP2 and Srebp1c in 3T3-CON, 3T3-WT and 3T3-A212P cells at the indicated time points during differentiation were assessed by real-time qPCR. Values were expressed as fold changes by normalizing to the level in control cells at Day 0. β-actin expression was used as an internal control. Data are presented as mean ± SEM. N = 3 independent experiments, each measured in triplicates. *p<0.05, **p<0.01, and ***p<0.001 vs. 3T3-CON cells at the same time points. (C) Expression of PPARγ in 3T3-CON, 3T3-WT and 3T3-A212P cells at day 6 was assessed by immuno-fluorescent staining using the anti-PPARγ antibody. DAPI was used for nuclear staining. Scale bar = 100 µm.

Previous studies, through the use of shRNA knockdown approaches, showed that PPARγ expression was regulated by Seipin [7], [13]. To test whether inhibition of adipogenesis by Seipin-A212P was associated with changes of PPARγ, we measured PPARγ expression in 3T3-WT, 3T3-A212P and 3T3-CON cells at various time points after DMI treatment. Consistent with normal adipocyte differentiation [17], [18], we observed that PPARγ expression peaked at 4 to 6 dpt in 3T3-WT and 3T3-CON cells. When compared with 3T3-WT and 3T3-CON cells, PPARγ expression was significantly lower at every time point in 3T3-A212P cells (Figure 2A and 2B). These results indicated that Seipin-A212P inhibited PPARγ expression and the inhibition occurred during the early stage of adipocyte differentiation. In addition, mRNA expression of Pref-1 and C/EBPβ, upstream adipogenic regulators of PPARγ and C/EBPα, was reduced in early days of adipogenesis in 3T3-A212P cells when compared to 3T3-CON cells (Figure S3), further demonstrating the potential role of Seipin during the early stage of adipogenesis.

Expression of adipogenic C/EBPα and PPARγ-target gene aP2 also significantly declined in 3T3-A212P cells from as early as 2 to 6 dpt (Figure 2B), supporting the notion that PPARγ activity was reduced in 3T3-A212P cells. We also measured the expression level of SREBP-1c, a key regulator of lipid synthesis. SREBP-1c expression was reduced in 3T3-A212P cells only after adipocyte differentiation had started at 2, 4 and 6 dpt, but not in pre-adipocyte stage of 0 dpt (Figure 2B), indicating that lipid synthesis necessary for the development of mature adipocytes was defective in 3T3-A212P cells. At 6 dpt, PPARγ was localized in the nuclei in 3T3-CON and 3T3-WT cells, while very low level of PPARγ was observed in the nuclei and cytoplasm in 3T3-A212P cells (Figure 2C). These results suggest that Seipin-A212P may inhibit adipogenesis and lipid synthesis by reducing PPARγ and SREBP-1c expression.

### Pioglitazone or Exogenous PPARγ Expression Partially Rescues Adipogenic Defect in Seipin-A212P Cells

**Figure 3:**
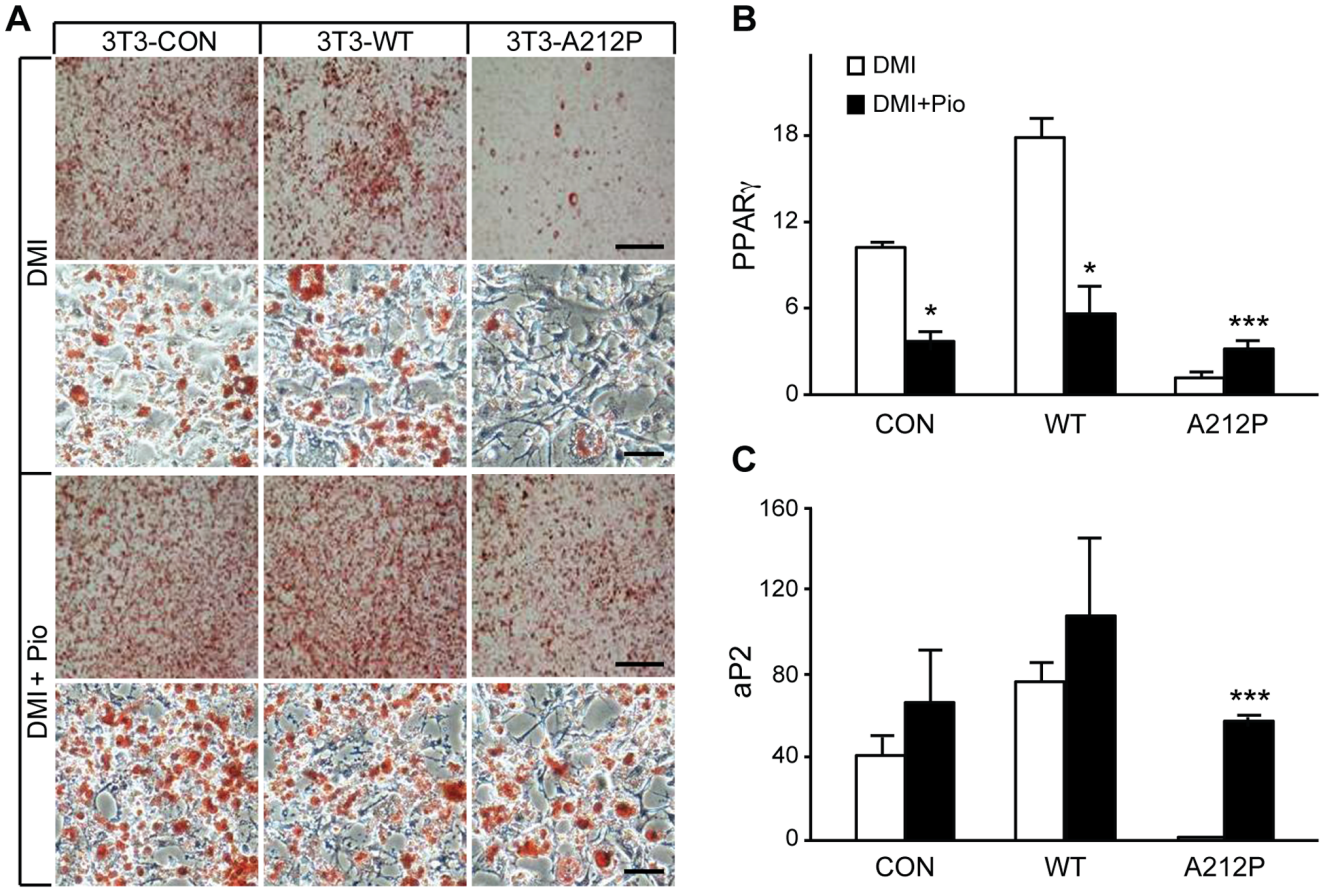
Pioglitazone (Pio) partially rescues defective adipogenesis in 3T3-A212P cells. (A) 3T3-CON, 3T3-WT and 3T3-A212P cells were treated with the standard adipogenic cocktail (DMI) or DMI plus 1 µM Pio. The cells were stained with Oil Red O at day 8 and photographed under a microscope. Scale bars = 200, 100, 200 and 100 µm, respectively. (B–C) Expression levels of PPARγ and aP2 were measured by real-time qPCR. β-actin expression was used as the internal control. Data are presented as mean ± SEM. N = 3 independent experiments, each measured in triplicates. *p<0.05 and ***p<0.001.

As PPARγ expression and activity were reduced in 3T3-A212P cells, we tested whether restoring PPARγ expression and activity could reverse the adipogenic defect in these cells. DMI-induced adipogenesis was severely inhibited in 3T3-A212P cells when compared with 3T3-CON and 3T3-WT cells (Figure 3A). However, when pioglitazone (Pio), a specific PPARγ agonist, was added to the induction medium, the adipogenic defect in 3T3-A212P cells was largely rescued, as evidenced by significantly more lipid accumulation than in the same cells under DMI treatment alone (Figure 3A and S4), consistent with a recent study [19]. The extent of lipid accumulation in Pio-rescued 3T3-A212P cells appeared comparable to 3T3-CON and 3T3-WT cells under DMI treatment alone (Figure 3A). As expected, treatment with DMI and Pio increased PPARγ expression in 3T3-A212P cells, as compared to those treated with DMI alone (Figure 3B). Co-treatment with DMI and Pio restored aP2 expression, indicating that the adipogenic defects in 3T3-A212P cells could be reversed by Pio-induced up-regulation of PPARγ activity (Figure 3C).

**Figure 4:**
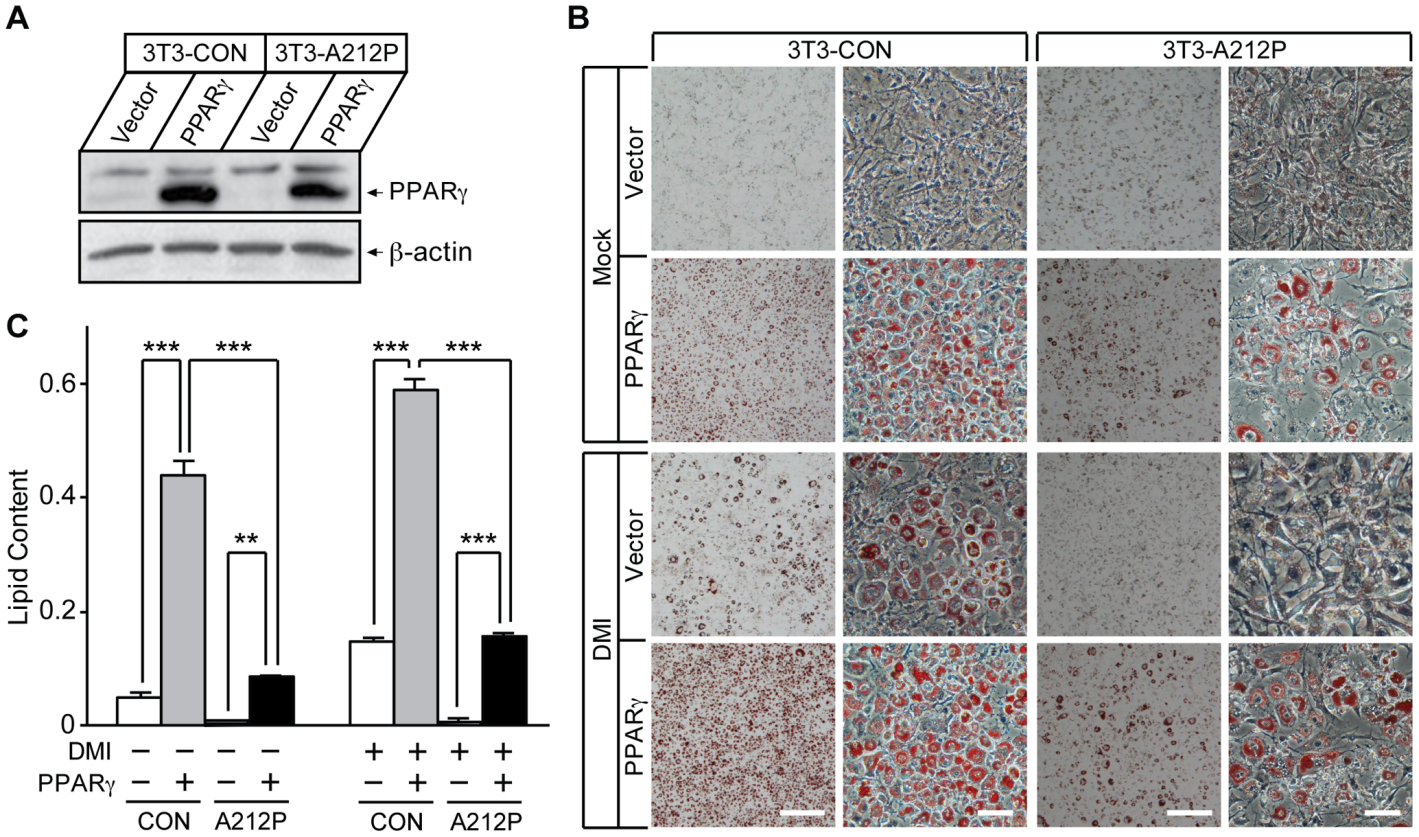
Exogenous expression of PPARγ partially rescues adipogenic defects in 3T3-A212P cells. (A) Western blot showing stable expression of exogenous PPARγ2 in 3T3-CON and 3T3-A212P cells. Lentivirus containing empty vector or PPARγ2 was transduced into 3T3-CON and 3T3-A212P cells. PPARγ2 (arrow) was detected by the PPARγ antibody. β-actin was used as a loading control. (B) At day 0, 3T3-CON and 3T3-A212P cells expressing PPARγ2 or control vector were treated with PBS (Mock) or the standard adipogenic cocktail containing dexamethasone, IBMX and insulin (DMI), followed by the standard differentiation protocol. At day 8, cells were stained with Oil Red O. Scale bars = 200, 50, 200 and 50 µm, respectively, and apply to each column of images. (C) Lipid content was measured for the cells in (B) after Oil Red O extraction with isopropanol. Data are presented as mean ± SD from three independent experiments. **p<0.01 and ***p<0.001.

To confirm that the rescue was by Pio-mediated up-regulation of PPARγ expression, we introduced an exogenous PPARγ gene or an empty vector into 3T3-CON and 3T3-A212P cells (Figure 4A), and tested adipogenesis and lipid accumulation in these cells. Under mock treatment, over-expression of PPARγ resulted in increased adipogenesis and lipid accumulation in 3T3-CON and 3T3-A212P cells (Figure 4B), although the adipogenic effect in 3T3-A212P cells was lower compared with that in 3T3-CON cells (Figure 4B). In the presence of DMI treatment, the extent of adipogenesis rescued by exogenous PPARγ expression in 3T3-A212P cells was comparable to that in 3T3-CON cells without exogenous PPARγ expression (Figure 4B). Lipid contents showed significant increase in 3T3-A212P cells expressing PPARγ when compared with those without exogenous PPARγ expression, under mock or DMI treatments, respectively (Figure 4C). Together, these results support the notion that increased PPARγ expression could account for the rescue by Pio, and demonstrate that increased PPARγ expression and activity can at least partially rescue the adipogenic defect in 3T3-A212P cells.

### Gene Expression Profile by Microarray Analysis in Seipin-A212P Mutant Cells

To examine the cellular changes underlying the adipogenic inhibition and PPARγ down-regulation in 3T3-A212P cells, we performed microarray analysis on 3T3-CON and 3T3-A212P cells at 4 dpt, when 3T3-A212P cells clearly displayed the adipogenic defects. We identified 14 genes down-regulated by >100 fold, and another 11 genes up-regulated by >30 fold in 3T3-A212P cells when compared with 3T3-CON cells (Table 1 and 2). Additionally, more than 1,700 and close to 1,800 genes were significantly down- and up-regulated, respectively (p<0.05, and fold changes >2, Table S1 and S2). As expected, a number of down-regulated genes were involved in fatty acid metabolism and LD formation, including *Thrsp, Gpd1* and *Cidec* (Table 1) [20], [21], [22], [23], [24]. Consistent with reduced adipogenesis, adipokine levels were also down-regulated, *e.g.* resistin was reduced by ∼1,300 fold, and adiponectin by ∼100 fold. It is worth noting that several genes involved in cytoskeleton maintenance, *Pkp3, Cyfip2* and *Mark1*, were up-regulated in 3T3-A212P cells, suggesting that cytoskeleton remodeling may play a role in the adipogenic process (Table 2) [25], [26], [27], [28]. In support of the notion, 3T3-A212P cells retained the fibroblast-like morphology (Figure 1C).

**Table 1:** Down-regulated genes in 3T3-A212P cells.

Gene symbol	Description	Fold Change	p-Value
Retn	Resistin (Retn)	1315	1.34E−04
Thrsp	Thyroid hormone responsive SPOT14 homolog	601	7.76E−04
Pygl	Liver glycogen phosphorylase	443	2.54E−04
Aatk	Apoptosis-associated tyrosine kinase	343	0.00106
Gpd1	Glycerol-3-phosphate dehydrogenase 1 (soluble)	282	0.00114
Cidec	Cell death-inducing DFFA-like effector c	232	5.82E−05
Sult1a1	Sulfotransferase family 1A, phenol-preferring, member 1	231	1.17E−05
Mrap	Melanocortin 2 receptor accessory protein	168	5.27E−05
Glut4	Solute carrier family 2 (facilitated glucose transporter), member 4	160	7.27E−05
Cfd	Complement factor D (adipsin)	132	1.06E−04
Adipoq	Adiponectin	105	1.45E−04
Sucnr1	Succinate receptor 1	104	1.87E−04
Nat8l	N-acetyltransferase 8-like	103	4.73E−04
Mgl1	Macrophage galactose N-acetyl-galactosamine specific lectin 1	102	0.00144

**Table 2:** Up-regulated genes in 3T3-A212P cells.

Gene symbol	Description	Fold Change	p-Value
Grem1	Gremlin 1, BMP antagonist	206	6.34E−04
Clec5a	C-type lectin domain family 5, member a, inflammation and immune response	82	0.00187
Akr1c18	Aldo-keto reductase family 1, member C18	79	0.00115
Scg5	Secretogranin V, ER protein as a molecular chaperone	70	9.51E−04
lgfbp5	Insulin-like growth factor binding protein 5	69	9.09E−04
Pkp3	Plakophilin 3, linking cadherins to intermediate filaments	48	0.004
EG637004	Predicted: similar to tissue-type vomeronasal neurons putative pheromone receptor V2R2	47	0.00141
Ankrd1	Ankyrin repeat domain 1, induced by IL-1 and TNF-alpha	42	0.00309
Cyfip2	Cytoplasmic FMR1 interacting protein 2, cell adhesion	42	9.40E−04
Mark1	MAP/microtubule affinity-regulating kinase 1	40	0.00333
Slc5a4	Solute carrier family 5, member 4, low affinity sodium-glucose cotransporter	31	0.00419

We performed qPCR on 15 target genes to validate the microarray analysis, and found consistent down- or up-regulation of genes at 4 dpt, similar to the data obtained from the microarray analysis (Table 3). Furthermore, up-regulated genes at 4 dpt exhibited similar changes even at the pre-adipocyte stage of 0 dpt (Table 3), suggesting that Seipin-A212P inhibits adipogenic and lipogenic genes at pre-differentiation or during the early stages of adipocyte differentiation.

**Table 3:** Validation of microarray analysis by real time qPCR.

	Day 4		Day 8
Gene name	Fold change by microarray	Fold change by qPCR	Fold change by qPCR
**DOWN-REGULATED GENES**			
Thyroid hormone responsive SPOT14 homolog (Thrsp)	601	250*	1
Apoptosis-associated tyrosine kinase (Aatk)	343	2*	1
Melanocortin 2 receptor accessory protein (Mrap)	168	14*	1.3
G0/G1 switch gene 2 (G0s2)	84	111***	1.2
PDZ domain containing 1 (Pdzk1)	79	33**	1.1
Transmembrane and coiled coil domains 3 (Tmcc3)	42	7*	5*
Kruppel-like factor 15 (Klf15)	29	33*	12*
PPARγ coactivator 1 beta (Ppargc1b)	19	8*	0.9
Melanocortin 2 receptor (Mc2r)	11	14*	32*
**UP-REGULATED GENES**			
Gremlin 1 (Grem1)	206	760*	13.9*
Insulin-like growth factor binding protein 5 (Igfbp5)	69	111*	32*
Matrix metallopeptidase 13 (Mmp13)	18	455**	118*
Interleukin 6 (Il6)	11	53*	13*
Kruppel-like factor 4 (Klf4)	4	8.6*	2.1*
Rho family GTPase 1 (RND1)	3	14***	2*

Microarray analysis was validated by real-time qPCR on 15 genes. mRNA levels at Day 0 and Day 4 during the standard differentiation procedure were compared between 3T3-A212P and 3T3-CON cells. Real-time qPCR was performed in 3 independent experiments, each in triplicates.

* p<0.05;

** p<0.01;

*** p<0.001 vs 3T3-CON cells.

### Inflammatory Responses in Seipin-A212P Cells

**Figure 5:**
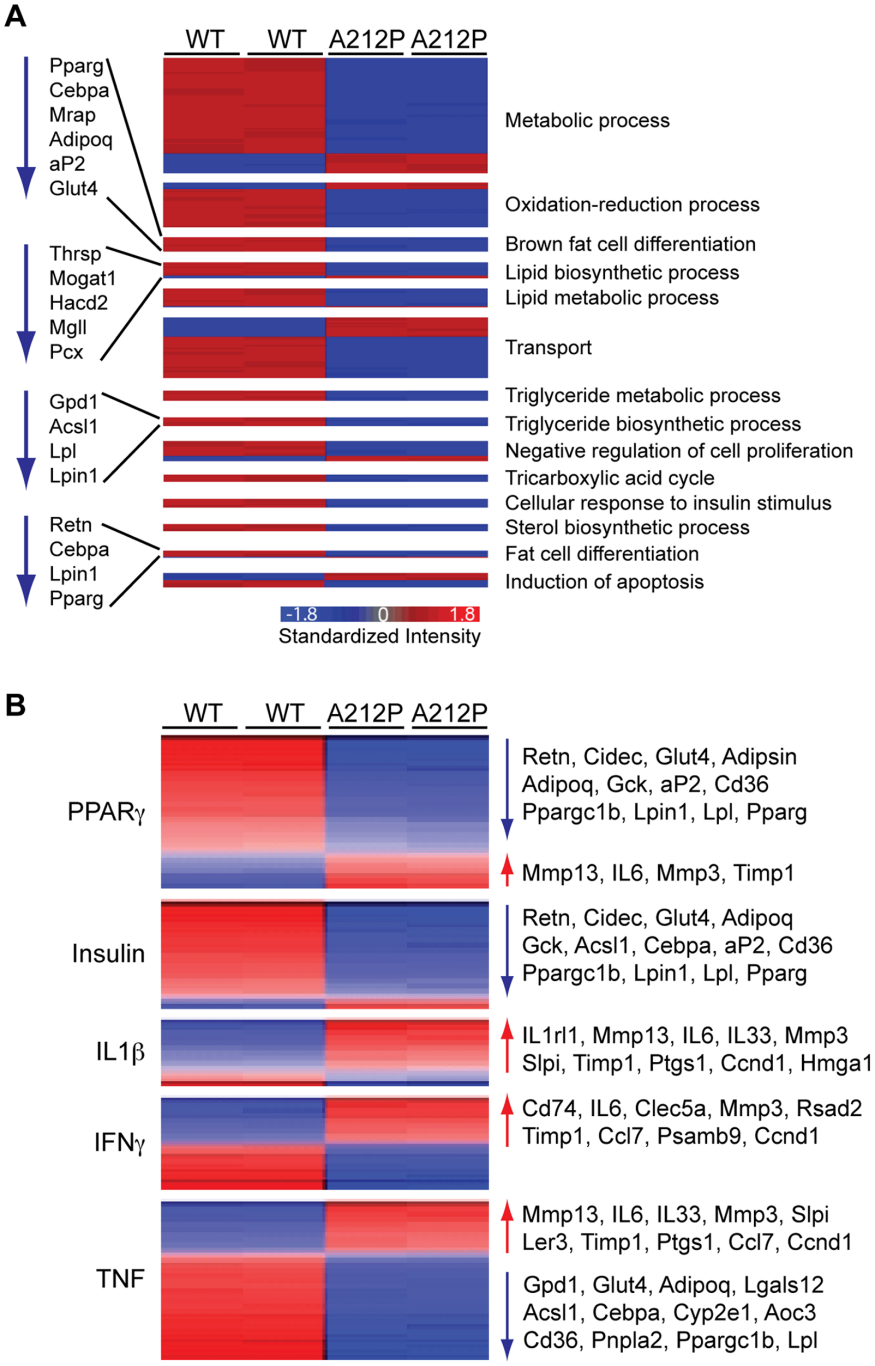
Gene expression profile in 3T3-A212P and 3T3-CON cells. (A) The heat map plots of the genes in the biological functions changed in the 3T3-A212P cells. The output of Agilent data was analyzed by using Partek Genome suite. The genes with at least 2 fold changes and p<0.05 were selected to run GO enrichment analysis. The biological function groups with no less than 5 genes and the enrichment score above 10 were clustered by heat map. (B) The networks of expression targets of PPARγ, Insulin, IL1β, IFNγ, TNF were identified by the SNEA program. The heat map images were generated by GenePattern software. Red and Blue denote genes with increased and decreased expression, respectively, in 3T3-A212P cells compared with the control cells.

To identify specific cellular processes and pathways that were affected in 3T3-A212P cells, we examined the cellular processes and signaling pathways by using the GO enrichment analysis of the Partek Genome Suite. Among the affected cellular processes in Seipin-A212P cells, significant reduction was observed in fat cell differentiation, and lipid and triglyceride metabolic and biosynthetic processes (Figure 5A). Other affected processes, including oxidation-reduction process and induction of apoptosis, may be secondary to the adipogenic defect instead of the direct consequence caused by Seipin-A212P mutation. The Sub-Network Enrichment Analysis (SNEA) program (Agilent) revealed the top ten affected pathways and the number of genes that were affected in respective pathways (Table 4). Key signaling pathways involved in adipogenesis and adipocyte functions, such as PPARγ (#1), Insulin (#6), PPARα (#9) and Leptin (#10) pathways, were down-regulated in 3T3-A212P cells; whereas pathways involved in inflammatory responses were up-regulated in 3T3-A212P cells, including TNF (#2), TNF family (#3), IFNγ (#4), IL1β (#7) and IL1 family (#8) pathways (Table 4). Many inflammatory response genes, including IL1r11, IL6, IL1α, IL13rα2, IL33, TNFsf18, TNFsf8, Jun-b and Jun, were up-regulated in 3T3-A212P cells, providing further support of increased inflammatory and stress responses (Table S3). The SNEA program further revealed the network of target genes regulated by PPARγ, TNF, IFNγ and IL1β (Figure 5B and Table S4, S5, S6, S7). Consistent with reduced PPARγ and insulin signaling, and increased inflammatory responses, most of the positively regulated target genes of PPARγ and insulin were down-regulated, while those of TNF, IFNγ and IL1β were up-regulated in 3T3-A212P cells (Figure 5B). Negatively regulated target genes of PPARγ, such as MMP3, MMP13, and IL6, were conversely up-regulated in 3T3-A212P cells (Figure 5B). Interestingly, some of these negatively regulated genes by PPARγ were also positively regulated by TNF, IFNγ and IL1β signaling (Figure 5B). In contrast, adipogenic genes PPARγ and C/EBPα were negatively regulated by TNF, IFNγ and IL1β pathways (Figure 5B). Together, these results suggest that inflammatory responses and the adipogenic process are intimately related, and that the inflammatory response may be the underlying mechanism for the defective adipogenic processes in 3T3-A212P cells.

**Table 4:** The top 10 affected pathways in 3T3-A212P cells.

No.	Pathway	Regulation	AffectedTargets	p-Value
1	Expression Targets of PPARγ	down	67	8.47E−14
2	Expression Targets of TNF	up	113	1.73E−13
3	Expression Targets of TNF family	up	89	3.29E−13
4	Expression Targets of IFNγ	up	95	1.79E−11
5	Expression Targets of SP1	up	109	8.94E−11
6	Expression Targets of Insulin	down	78	2.66E−10
7	Expression Targets of IL1β	up	71	3.51E−10
8	Expression Targets of IL1 family	up	59	7.97E−10
9	Expression Targets of PPARα	down	51	8.31E−10
10	Expression Targets of Leptin	down	48	9.08E−10

### Seipin-A212P Induces Inflammatory Responses and Inhibits Adipogenesis at Pre-differentiation and Early Stages of Differentiation

In the RT-qPCR validation experiments, we found that some of the genes, including the inflammatory IL6, were affected even at pre-differentiation 0 dpt (Table S3). To test whether the inflammatory response could be activated before adipocyte differentiation, we measured gene expression of inflammation markers in 3T3-CON and 3T3-A212P pre-adipocytes. All of the tested markers, including IL6, iNOS, TNFα, IL1β, TNFβ, MCP1, COX2, LXRα and SRC1 were up-regulated by at least 2 fold in 3T3-A212P cells, when compared with 3T3-CON cells (Figure S5). These results show that Seipin-A212P could induce an inflammatory response in pre-adipocytes. Considering the close association of the inflammatory response and adipogenesis, it is tempting to propose that expression of Seipin-A212P at pre-differentiation and early stages of post-differentiation may inhibit adipogenic processes by inducing an inflammatory response.

**Figure 6:**
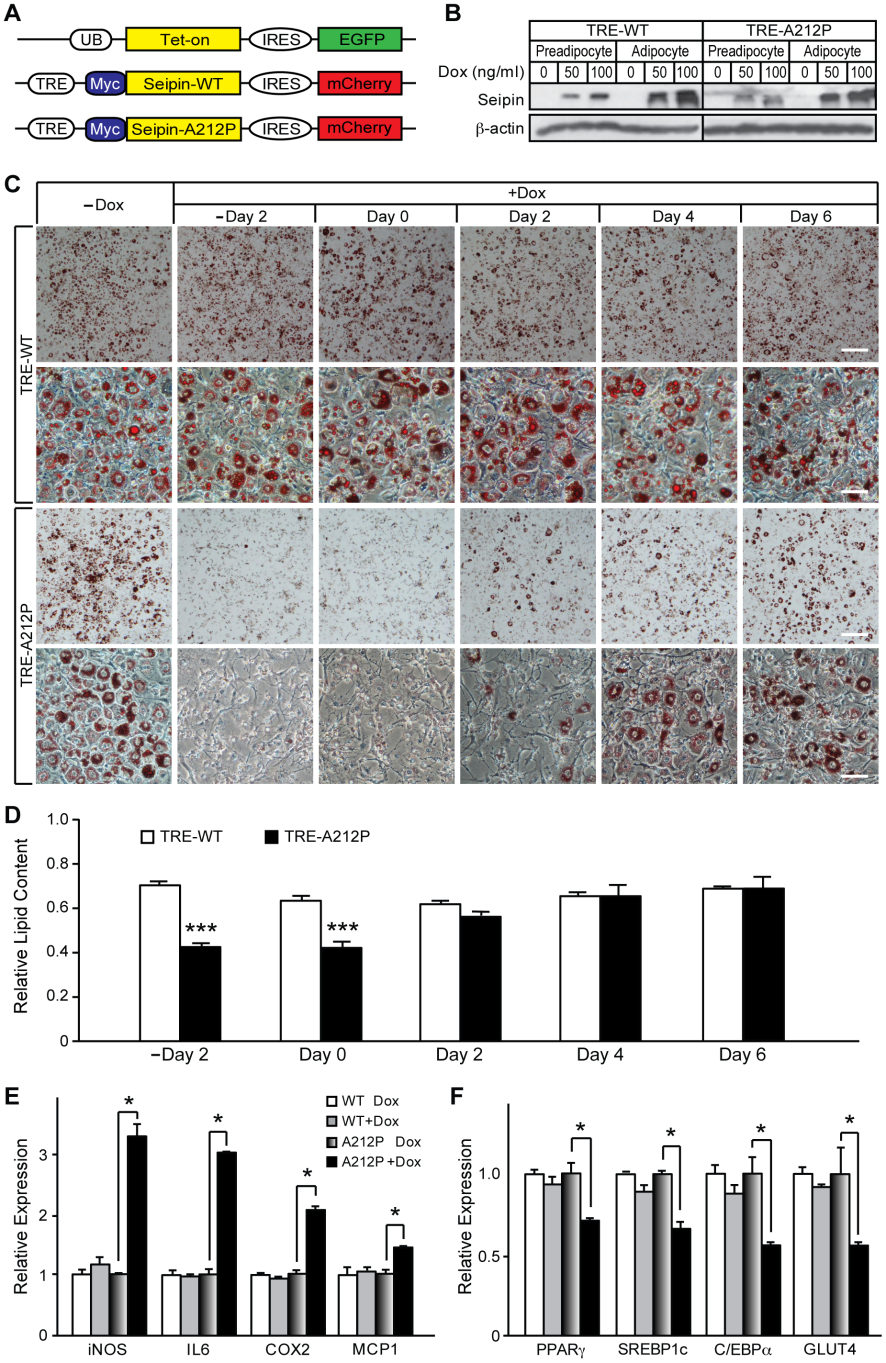
Seipin-A212P inhibits adipogenesis at early stages of adipocyte differentiation. (A) A schematic diagram of the constructs used in generating the Tet-on inducible cell lines. Both mouse Seipin wild type (Seipin-WT) and A212P Seipin mutant (Seipin-A212P) were tagged with Myc at the N-terminus, and co-expressed with mCherry. Expression of Seipin and mCherry was under the control of the TRE promoter, which was activated by the Tet-on protein in the presence of doxycycline (Dox). 3T3-L1 cells were first infected with virus co-expressing the Tet-on protein and EGFP to create Tet-on stable cells. These cells were then infected with virus containing TRE-WT or TRE-A212P to generate the 3T3-TRE-WT and 3T3-TRE-A212P inducible cell lines, respectively. (B) Tight regulation of Seipin expression by Dox treatment in 3T3-TRE-WT and 3T3-TRE-A212P cells at pre-adipocyte and mature adipocyte stages. Higher Seipin expression was achieved with increased amount of Dox treatment. Seipin was detected by Myc antibody. β-actin was used as a loading control. (C) 3T3-TRE-WT and 3T3-TRE-A212P cells were treated with 100 ng/ml of Dox at the indicated time points during adipocyte differentiation before they were stained with Oil Red O and photographed under a microscope at day 10. Scale bars = 200, 50, 200 and 50 µm, respectively, and apply to each row of images. (D) Lipid contents of 3T3-TRE-WT and 3T3-TRE-A212P cells were quantified by using Oil Red O extraction and normalized to the same type of cells without Dox treatment. Data are presented as mean ± SD from three independent experiments. ***p<0.001 vs. TRE-WT cells that were treated with Dox on the same day. (E-F) Expression of various inflammatory response markers (E) and adipogenic genes (F) were measured by real-time qPCR in 3T3-TRE-WT and 3T3-TRE-A212P cells treated with or without 100 ng/ml Dox at minus day 2 and harvested at day 4 post differentiation. Data are presented as mean ± SD from three independent experiments. Comparison was made between the same type of cells with or without Dox treatment. *p<0.05.

To test this hypothesis, we first generated stable Tet-on inducible cell lines expressing Seipin wild type (TRE-WT) or A212P mutant (TRE-A212P) (Figure 6A). In TRE-WT and TRE-A212P cells, expression of Seipin-WT and Seipin-A212P could be tightly controlled and efficiently induced by doxycycline (Dox) treatment in pre-adipocytes and mature adipocytes, and no Seipin expression was observed without Dox treatment (Figure 6B and S6). We then induced expression of Seipin-WT or Seipin-A212P in TRE-WT and TRE-A212P cells at various time points of adipocyte differentiation, and examined their expression on adipocyte differentiation and lipid accumulation. As expected, both TRE-WT and TRE-A212P cells differentiated into normal lipid-filled adipocytes in the absence of Dox treatment (Figure 6C). Consistent with the findings in 3T3-WT cells, TRE-WT cells exhibited normal adipocyte differentiation and lipid accumulation regardless of when Seipin-WT expression was induced (Figure 6C and 6D). In contrast, adipogenesis and lipid accumulation were impaired when TRE-A212P cells were treated with Dox at pre-differentiation and early stages of differentiation, but not at 2 dpt or later (Figure 6C and 6D). Together, these results demonstrate that inhibition of adipogenesis by Seipin-A212P occurred during the initial stages of adipocyte differentiation.

We next examined whether expression of Seipin-A212P at pre-differentiation was associated with activation of inflammatory responses in TRE-A212P cells. Expressions of inflammatory response markers, such as iNOS, IL6, COX2 and MCP1, were significantly elevated (Figure 6E). In contrast, Dox-induced expression of Seipin-WT at the same time point did not result in an apparent inflammatory response (Figure 6E). Furthermore, expressions of PPARγ, SREBP1c, C/EBPα and GLUT4 were significantly down-regulated in Dox-treated TRE-A212P cells, but not TRE-WT cells (Figure 6F), consistent with the earlier observation that expressions of adipogenic regulators and markers were down-regulated during the early stages of adipocyte differentiation in 3T3-A212P cells (Figure 2B). These findings, together with the microarray and RT-qPCR analyses, suggest that Seipin-A212P acts during the early stages of adipocyte differentiation to inhibit adipogenesis, possibly via the Seipin-A212P-induced inflammatory response.

### Seipin-A212P Expression Activates an Inflammatory Response and is Associated with ER Stress Response

**Figure 7:**
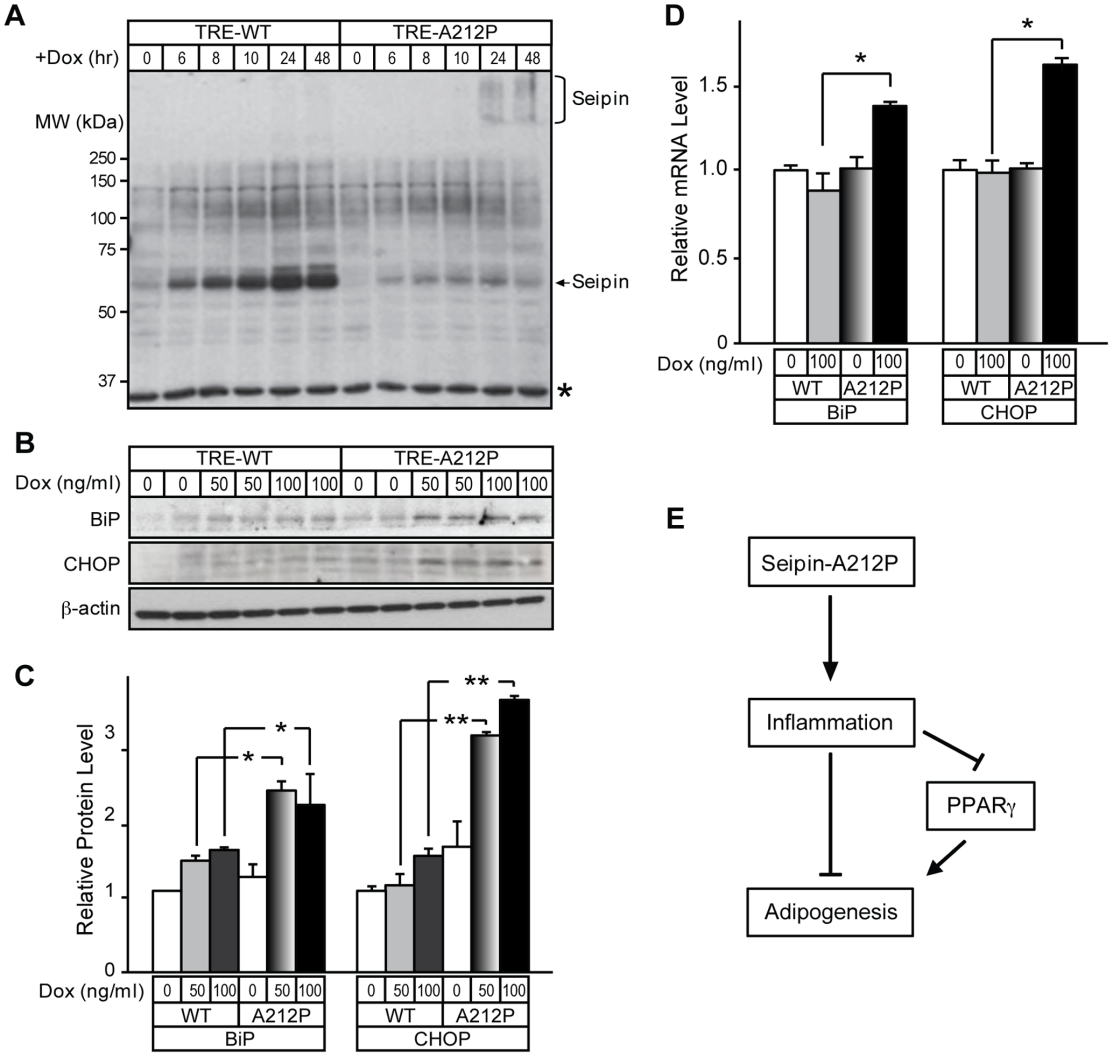
ER stress response in 3T3-A212P pre-adipocytes. (A) 3T3-TRE-WT and 3T3-TRE-A212P cells were treated with 100 ng/ml of Dox at the indicated time points. Total cell lysate was collected and immuno-blotting was performed by using the Myc antibody. The asterisk indicates a nonspecific band as a loading control. (B) 3T3-TRE-WT and 3T3-TRE-A212P cells were treated with 50 or 100 ng/ml Dox after cells reached 100% confluency. Two days later, total cell lysate was collected and immuno-blotting was performed using BiP and CHOP antibodies. β-actin was used as the loading control. (C) Quantification of protein expression levels in (B). Data are presented as mean ± SD from a representative of six independent experiments. Comparison was made between 3T3-TRE-WT and 3T3-TRE-A212P cells receiving the same dosage of Dox treatment. *p<0.05, **p<0.01. (D) 3T3-TRE-WT and 3T3-TRE-A212P cells were treated with 100 ng/ml Dox after cells reached 100% confluency. Two days later, expression levels of ER stress markers BiP and CHOP were measured by real-time qPCR. Data are presented as mean ± SD from three independent experiments. Comparison was made between 3T3-TRE-WT and 3T3-TRE-A212P cells receiving the same dosage of Dox treatment. *p<0.05, **p<0.01. (E) A proposed model on how Seipin-A212P inhibits adipogenesis. Seipin-A212P induction is associated with inflammatory responses in the early predifferentiation stage, which in turn inhibits PPARγ expression and suppresses adipogenesis.

Numerous studies have shown a close relationship between the inflammatory response and ER stress, including a recent report that indicated a direct link between the two processes [29]. Since an inflammatory response was observed in 3T3-A212P and TRE-A212P cells, we decided to test whether ER stress was also present in these cells. We examined the protein expression pattern in TRE-WT and TRE-A212P cells. Seipin-WT was mostly present as a monomer, and its expression increased over time. In contrast, the monomeric form of Seipin-A212P showed a slight increase up to 24 hours after Dox treatment, and the expression level decreased considerably at 48 hours after Dox treatment. Concurrently, at 24 hours and 48 hours after Dox treatment, the expression levels of the high molecular form of Seipin-A212P increased (Figure 7A). To test whether the low protein level and aggregation of Seipin-A212P could be attributed to unfolded protein response (UPR), we examined expression levels of ER stress markers, BiP and CHOP [30]. The expression levels of BiP and CHOP were increased in TRE-A212P, but not TRE-WT cells after Dox treatment (Figure 7B and C), suggesting that induced expression of Seipin-A212P was associated with increased levels of BiP and CHOP. Consistent with increased protein levels, the transcripts of BiP and CHOP were also increased in Dox-treated TRE-A212P cells (Figure 7D).

### Inhibition of Inflammation Restores Adipogenesis in Seipin-A212P Expressing Cells

**Figure 8:**
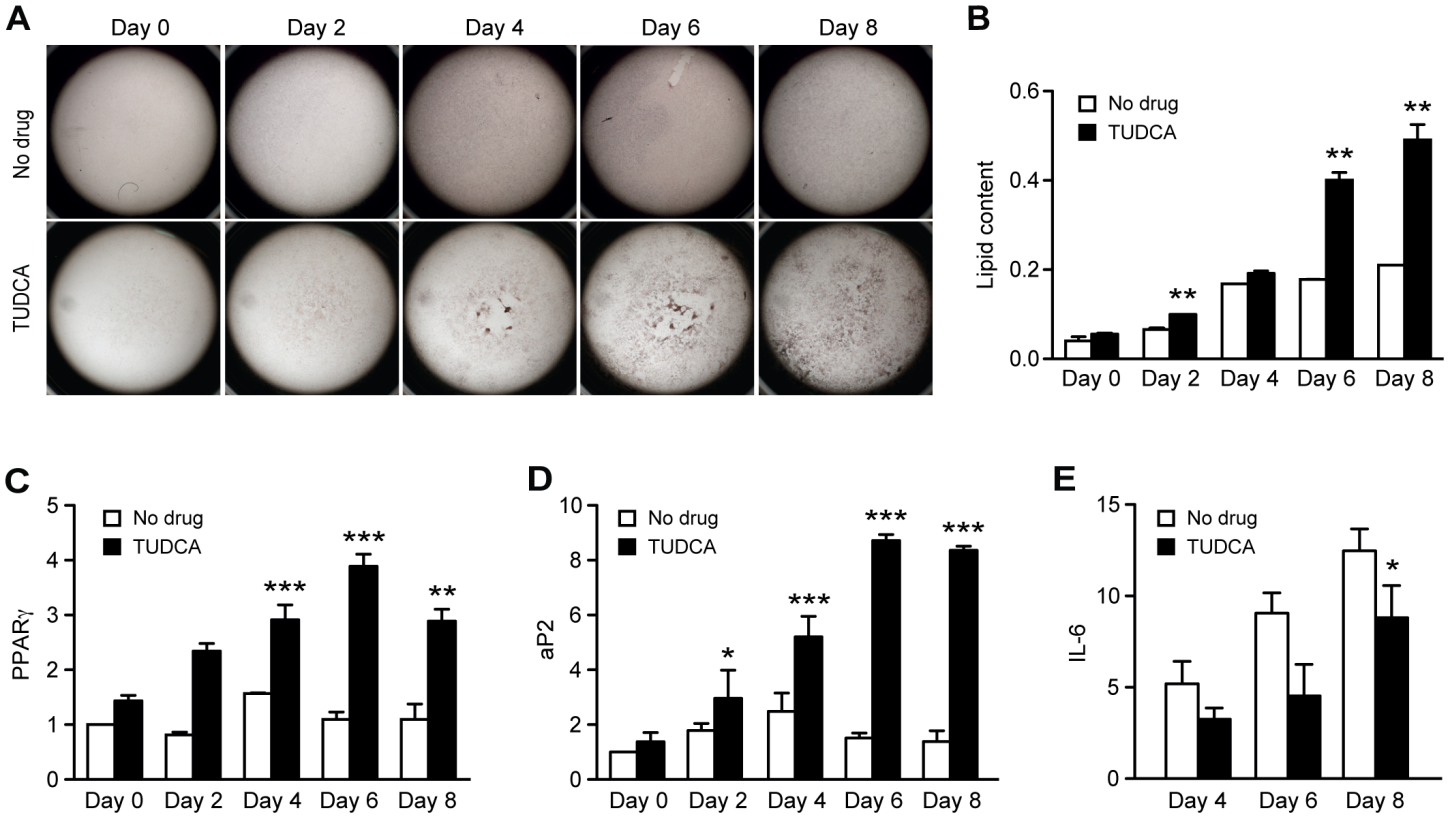
TUDCA treatment rescues adipogenic defect in 3T3-A212P cells. 3T3-A212P cells were pre-treated with or without 1 mg/ml of TUDCA two days before initiating adipogenic differentiation with DMI. Cells were subsequently collected for Oil Red-O staining or RNA extraction. (A) Oil Red O staining and (B) Oil Red O extraction of cells at the indicated differentiation time points with or without TUDCA treatment. (C–E) Cells treated with or without TUDCA were subjected to RNA extraction and detected for expression of adipogenic markers PPARγ (C) and aP2 (D) and inflammatory marker IL-6 (E) at the indicated differentiation time points.

Since Seipin-A212P expression led to enhanced activation of inflammatory markers (Figure 5, 6E and S5), we examined if inhibition of inflammation could rescue the adipogenic defect in Seipin-A212P expressing cells. Treatment with bile acid and chemical chaperone TUDCA resulted in a dramatic improvement of adipogenesis in 3T3-A212P cells, as evidenced by Oil Red O staining, lipid quantification and elevated expression of PPARγ and aP2 (Figure 8). Importantly, this adipogenic rescue was associated with reduced IL6 expression (Figure 8C). Additionally, treatment with the anti-inflammatory indomethacin significantly rescued the adipogenic defect in 3T3-A212P (Figure S7). Together, these results show that expression of Seipin-A212P is associated with the inflammation-induced adipogenic defects and that pharmacological inhibition of inflammation through TUDCA or indomethacin counteracts this effect and restores adipogenesis.

## Discussion

Seipin A212P mutation was identified and associated with lipodystrophic phenotype of CGL2. While loss of function mutations by deletion or knockdown of Seipin has been studied, the role of the missense A212P in the pathogenesis of lipodystrophy is still unclear [7], [13], [14], [31]. It has yet to be determined whether the A212P mutation leads to partial or complete loss of protein function. Our current study demonstrates that expression of Seipin-A212P, but not non-lipodystrophic Seipin-N88S or Seipin-S90L, results in dramatically impaired adipogenesis in 3T3-L1 cells, and this inhibition is associated with activation of inflammatory pathways. The distinct functional consequence of the Seipin mutants in adipogenesis is consistent with a recent study, which reported that overexpression of N88S and S90L mutants, but not A212P, produced a number of fatty acid-induced small lipid droplet clusters in non-adipogenic HeLa and NIH3T3 cells [32]. However, the same study identified that overexpression of Seipin-WT reduced formation of lipid droplets, whereas no adipogenic defects were observed in our study. A potential explanation is that we selected by FACS the population of cells with only moderate overexpression of proteins in this study, thus avoiding overexpression artefacts. In addition, Seipin functions differently in non-adipocytes from adipocytes. Consistent with this notion, a recent study in *Drosophila* demonstrates a tissue-specific role of Seipin in promoting lipid storage in adipogenic tissue while preventing lipid accumulation in other tissues [14].

Previous studies have shown that Seipin null mutation affects phospholipid synthesis in yeast and fibroblasts of human lipodystrophic patients, suggesting that components of the phosphatidic acid pathway may function as a regulator of PPARγ and subsequently adipogenesis [6], [12], [33], [34]. Similar to the findings for the null mutation, we found reduced PPARγ expression in 3T3-A212P cells. The inhibitory effect of Seipin-A212P occurred during the early stages of adipocyte differentiation. The negative regulation of both PPARγ expression and adipogenesis in 3T3-A212P cells could be partially rescued by treatment with the PPARγ agonist pioglitazone or through overexpression of PPARγ, indicating that the adipogenic inhibition by Seipin-A212P was at least partially through the down-regulation of PPARγ expression. In Pio-treated 3T3-CON and 3T3-WT cells, we noticed decreased PPARγ expression despite moderately increased adipogenesis (Figure 3B). The paradoxical observation was also reported in previous reports, which showed the inhibitory effect of Pio and rosiglitazone on PPARγ expression, but not on its activity, in 3T3-L1 cells [35], [36]. As expected, overexpression of exogenous PPARγ in 3T3-CON cells dramatically enhanced adipogenesis and lipid storage (Figure 4). These results imply that Seipin may have a regulatory role upstream of PPARγ along the same signaling cascade, making functional Seipin necessary for maximal expression and adipogenic activity of PPARγ, and that Seipin may affect other adipogenic molecules that are not regulated by PPARγ. Further studies are needed to define the molecular pathways underlying the PPARγ-independent defect.

While the precise mechanism underlying the adipogenic defect and down-regulation of PPARγ expression by Seipin-A212P remains unclear, our findings of concomitant elevation in inflammation markers suggest that inflammatory responses may play an important role in the adipogenic defect. A number of studies have linked the inflammatory response with adipogenic inhibition [37], [38], [39]. In adipocytes, disruption of inflammatory genes IKKβ, JNK and IKKε has the potential to promote adiposity and subsequently reverse the obesity-induced insulin resistance [40], [41], [42], while up-regulation of adipocytokines, such as TNFα, IFNγ, IL1, IL6, TGFβ and leukemia inhibitory factor (LIF) has been postulated to inhibit adipogenesis and/or promote lipolysis through regulation of PPARγ. In contrast, PPARγ agonists promote adipocyte differentiation and insulin sensitivity, possibly by inhibiting TNFα and other inflammatory adipocytokine production [43], [44]. Consistent with these studies, we found that various proinflammatory markers, including TNFα, IL6, iNOS, COX2 and MCP-1, were up-regulated during the pre-adipocyte stage in both 3T3-TRE-A212P and 3T3-A212P cells. The up-regulation of inflammatory markers in these cells is associated with reduced PPARγ and its target gene expression, and inhibition of adipogenesis.

TUDCA, a chemical chaperone, has been reported to exhibit a variety of effects including anti-inflammatory, anti-apoptotic or as an ER stress reliever [45], [46], [47]. Accordingly, we observed downregulation of IL6 and significant rescue in the adipogenesis defect of 3T3-A212P when treated with TUDCA (Figure 8). In addition, the rescue could also be achieved with indomethacin (Figure S7), which was previously reported to downregulate IL6 [48], [49], [50]. Thus the inflammatory response associated with Seipin-A212P may be a key underlying mechanism for the adipogenic defects in 3T3-A212P and 3T3-TRE-A212P cells.

In obese subjects, macrophage infiltration in adipose tissue plays an important role in inducing the inflammatory response and ultimately contributes to the development of obesity-linked insulin resistance and metabolic syndrome [38], [51], [52]. Our study presents evidence that ER stress may potentially be a link between Seipin-A212P and the inflammatory response in 3T3-A212P cells. Chen *et al.* recently reported that mouse embryonic fibroblasts isolated from Seipin^−/−^ mice did not exhibit increased ER stress markers and their adipogenic defect was not fully rescued by pioglitazone [53]. This is in contrast with the previous result by the same group that the adipogenic defect of 3T3-L1 cells by Seipin knockdown was almost fully restored by treatment with pioglitazone at early stages [13]. Whether the discrepancy arises from differences in cell types, experimental setups, or methods of disrupting Seipin functions requires further investigation. UPR/ER stress response leads to activation and secretion of adipocytokines such as TNFα and IL6 in both adipose tissue and the 3T3-L1 cells [54], [55], [56]. The secreted adipokines subsequently recruit peripheral macrophages leading to an inflammatory response *in vivo* [57]. Systemic inflammation and massive macrophage infiltration in white and brown adipose tissue depots were observed in a lipodystrophic mouse model with nSREBP-1c overexpression under the aP2 promoter [58]. In light of these observations, our current study offers a potential mechanistic model in the understanding of lipogenic defects associated with Seipin-A212P, which is linked to induction of an inflammatory response (Figure 7E). Our study thus suggests reducing inflammation as a potential treatment strategy for lipodystrophy in patients with *BSCL2/Seipin* mutations.

## Materials and Methods

### Cell Culture

The 3T3-L1 cell line (American Type Culture Collection, Manassas, VA) was maintained in high glucose DMEM supplemented with 10% FCS (Gibco BRL) and 1% penicillin/streptomycin. 3T3-L1 pre-adipocytes were seeded in 6-well-plates and adipocyte differentiation was induced 2 days after cells reached confluence (Day 0) in high glucose DMEM supplemented with 10% FCS and adipogenic cocktail containing 1 µM Dexamethasone (Dex), 0.5 mM isobutylmethylxanthine (IBMX) and 10 µM insulin. Forty-eight hours later (Day 2), cells were switched to high glucose DMEM supplemented with 10% FCS and 1 µM insulin. Thereafter, media were replaced with high glucose DMEM plus 10% FCS every 2 days. 293TN cells were grown in high-glucose DMEM supplemented with 10% FCS and 1% penicillin/streptomycin. All cells were maintained at 37°C in a 5% CO_2_ humidified incubator.

### Plasmid Constructs

Lentiviral plasmids expressing murine Seipin WT (Seipin-WT), Seipin A212P mutant (Seipin-A212P), Seipin N88S (Seipin-N88S), Seipin S90L (Seipin-S90L), α-synuclein WT (α-synuclein-WT), α-synuclein A30P (α-synuclein-A30P) and PPARγ2 were made by subcloning the PCR products containing the full-length coding region of Seipin-WT, Seipin-A212P, Seipin-N88S, Seipin-S90L, α-synuclein-WT, α-synuclein-A30P [59], or PPARγ2 downstream of the ubiquitin promoter in a lentiviral shuttle vector containing an IRES-EGFP cassette. The full length Seipin cDNA sequence reference number was BC061689, and the vector containing full length Seipin was ATCC-10470632. Lentiviral plasmids for the Tet-Advanced Expression Systems were constructed by subcloning the tetracycline-controlled transcriptional transactivator gene with its upstream promoter from the pTet-on-Advanced Vector (Clontech) into a lentiviral shuttle vector. Seipin-WT and Seipin-A212P were cloned into pTRE-Tight vector (Clontech) first, and then the fragment containing the TRE-Tight promoter and the Seipin-WT or Seipin-A212P gene were excised and ligated into a lentiviral shuttle vector containing an IRES-mCherry cassette. Primers and detailed cloning strategies are available upon request.

### Lentiviral Infections

The lentiviral shuttle vectors containing the target genes and three “helper” plasmids (encoding HIV-1 gag-pol, HIV-1 rev, and VSV-G envelope) were co-transfected into the 293TN cell line according to established procedures [60]. Lentiviral plasmids carrying Myc-tagged Seipin cDNA (Seipin-WT, Seipin-N88S, Seipin-S90L, Seipin-A212P, α-synuclein-WT, or α-synuclein-A30P), an internal ribosome entry site (IRES) and EGFP coding region (Figure 1A) were co-transfected with helper plasmids into 293TN cells to produce lentiviral particles for subsequent infection of 3T3-L1 cells. Twelve to fourteen hours after transfection, the medium was removed and changed to fresh medium. Medium containing the virus particles were then harvested after 24 hrs and filtered through 0.45-µm filters. Virus containing medium along with 8 µg/ml of Polybrene (Sigma) were added to 3T3-L1 cells for overnight infection, and the medium was replaced with fresh 10% FCS/DMEM (v/v) the following day. Two days after infection, the medium was changed and the cells were further passaged before fluorescence sorting by FACS to establish the stable cell lines. As a control, we also generated a stable 3T3-L1 cell line carrying only the IRES and EGFP sequences (3T3-CON). We selected stable cell lines at relatively low fluorescence levels by FACS to avoid high overexpression artifacts. To establish the Tet-on inducible 3T3-L1 cell line, virus expressing the Tet-Advanced transactivator gene and TRE-Tight-Seipin was added simultaneously to the cells in a 1∶1 ratio.

### Real Time qPCR

This was performed essentially as previously described [61]. For a detailed description, refer to Supporting Information. In brief, total RNA from cell culture was extracted using Trizol reagent (Invitrogen) and treated with DNase I prior to cDNA conversion using the RevertAid H minus first strand cDNA synthesis kit (Fermentas, USA) with oligo d(T) 18 primer according to manufacturer’s instructions. For quantitative PCR, cDNA samples were analysed in triplicates using the SYBR® Green PCR Master Mix reagent kit (Applied Biosystems) on a StepOnePlus™ Real-Time PCR System (Applied Biosystems). Relative mRNA levels were calculated and normalized to GAPDH.

### Immuno-blotting and -fluorescence

Cell lysates were prepared either in 1x SDS loading buffer or standard RIPA lysis buffer. Protein quantifications from RIPA lysis buffer treatment were determined by using the Bradford Protein Assay (BioRad). Equal amounts of protein (30 µg) were loaded into 12% SDS-PAGE mini gels and blotted onto a nitrocellulose membrane by using the iBlot Dry Blotting System (Invitrogen). Membranes were either blocked in 5% non-fat milk or 5% BSA for 1 hr at room temperature. They were then probed with primary antibodies in the same buffer either for 1 hr at room temperature or overnight at 4°C, followed by HRP-conjugated secondary antibodies. Primary antibodies used were anti-Myc (Roche), anti-PPARγ (Cell Signaling), anti-BiP (Cell Signaling), anti-GADD153/CHOP (Santa Cruz) and anti-β-Actin (Santa Cruz). For immuno-fluorescence, cells were cultured on polylysine-coated glass coverslips, fixed with 4% paraformaldehyde for 10 min, permeabilized with PBS containing 0.5% triton X-100 for 10 min, and blocked in ICC buffer (3% BSA, 3% goat serum, and 0.15% triton X-100 in PBS) for 1 hr at room temperature. Cells were then probed with antibodies against Myc (Santa Cruz) or PPARγ, followed by fluorescence-conjugated secondary antibodies (Invitrogen). The coverslips were mounted onto slides with Vectashield mounting medium containing 4′6-diamidino-2-phenylindole (Vector Laboratories). Images were acquired by using a Nikon confocal microscope (Nikon).

### Oil Red O Staining and Isopropanol Extraction

Oil Red O staining was performed as described [62]. The stained cells were first imaged on Nikon SMZ1500 and TS100 microscopes before they were air-dried and stained lipids were extracted by using isopropanol for lipid content measurements. The extracted solution was measured at OD500 wavelength as the relative cellular lipid content. After extraction, cells were lysed in 1× SDS loading buffer and immunoblotting was performed to detect β-actin. Lipid content was then normalized to β-actin level.

### Microarray Analysis

RNA samples for microarray analysis were prepared from the 3T3-CON and 3T3-A212P cells at day 4 after standard adipogenic cocktail treatment. Cells from each group were examined in duplicate. Total RNA was extracted by the RNeasy kit (Qiagen). The integrity of the RNA was confirmed by using the RNA 600 LabChip™ kit on an Agilent 2100 bioanalyzer. 200 ng RNA and 2 µl (1∶5,000 dilution) Agilent One-Colour Spike-In mix were labelled with the Agilent Low Input Linear Amplification Kit PLUS (Agilent Technologies UK) according to the manufacturer’s instruction. Briefly, cDNA was synthesized for 2 hr at 40°C followed by denaturation for 10 min at 65°C. The synthesis of the fluorescent-labelled cRNA was performed during the second incubation step (2 h at 40°C). The labelled cRNA was purified with the RNeasy Mini Kit (Qiagen) according to the manufacturer’s protocol. The cRNA was hybridized to Agilent mouse 4×44,000 element DNA microarrays (G4846A) in a reference-based design (Agilent Technologies UK). Hybridization was performed according to the manufacturer’s protocol (using 2 µg of the labeled sample as input). The slides were scanned with the Agilent G2565BA Microarray Scanner System. The Agilent G2567AA Feature Extraction Software v.9.1 (Agilent Technologies UK) was used for data extraction and quality control. Extracted data were analyzed using GeneSpring GX 7.3.1(Silicon Genetics, CA, USA). Agilent standard scenario normalizations for FE (1-colour) arrays were applied to all data sets. Relative expression of each probe in 3T3-CON and 3T3-A212P cells was determined; probes differentially expressed by greater than 2.0-fold (p<0.05) were selected as shown in Table S1 and S2. GO enrichment analysis of the Partek Genome Suite and the Sub-Network Enrichment Analysis (SNEA) program (Agilent) was utilised to assemble functional networks associated with the differentially expressed probes.

### Statistical Analysis

Comparisons of data were made by using two-tailed unpaired t-test with equal variance or ANOVA followed by Tukey’s post hoc test. Statistical significance limit was displayed as *p<0.05, **p<0.01 or ***p<0.001.

## Supporting Information

Figure S1 **Normal cell viability in 3T3-A212P cells.** 3T3-CON and 3T3-A212P cells were grown to full confluency and subsequently subjected to standard DMI cocktail. Fresh medium were added every 2 days and cells were trypsinized and collected at day 8. Cell count and cell viability were then performed using the Countess Automated Cell Counter according to the manufacturers instructions. Data are presented as mean ± SEM. N = 3 independent experiments, each measured in triplicates.
(TIF)

Figure S2 **Overexpression of misfolding proteins does not inhibit adipogenesis.** Stable 3T3-L1 cells expressing Seipin-N88S, Seipin-S90L, α-synuclein-WT or α-synuclein-A30P were grown to full confluency and subsequently subjected to standard DMI cocktail. Cells were then collected and subjected to Oil Red-O staining and extraction at the indicated time points. Data are presented as mean ± SEM. N = 2 independent experiments, each measured in duplicates. **p<0.01 and ***p<0.001.
(TIF)

Figure S3 **Early adipogenic markers are down-regulated in 3T3-A212P cells.** Expression levels of Pref-1 and C/EBPβ in 3T3-CON and 3T3-A212P cells at the indicated time points during differentiation were assessed by real-time qPCR. Values were expressed as fold changes by normalizing to the level in control cells at Day 0. β-actin expression was used as an internal control. Data are presented as mean ± SEM. N = 3 independent experiments, each measured in triplicates. *p<0.05, **p<0.01, and ***p<0.001 vs. 3T3-CON cells at the same time points.
(TIF)

Figure S4 **Dose-dependent rescue of adipogenic defect by pioglitazone in 3T3-A212P cells.** 3T3-L1, 3T3-CON and 3T3-A212P cells were grown to full confluency and subsequently subjected to standard DMI cocktail with pioglitazone at the indicated concentrations. Pioglitazone was included throughout differentiation steps at the same concentrations. Cells were then collected for Oil Red-O staining and extraction at day 8. Data are presented as mean ± SEM. N = 3. *p<0.05, **p<0.01, and ***p<0.001.
(TIF)

Figure S5 **Seipin-A212P induces an inflammatory response in pre-adipocytes.** At the pre-adipocyte stage, the total RNA of 3T3-CON and 3T3-A212P was extracted and expression of various inflammation response genes assessed by real-time qPCR. mRNA levels of different inflammation response markers were compared between 3T3-CON (white bar) and 3T3-A212P (black bar) cells. Data are presented as mean ± SD from three independent experiments. *p<0.05.
(TIF)

Figure S6 **Induction of Seipin-WT and Seipin-A212P expression in the Tet-inducible stable cell lines**. At the pre-adipocyte and mature adipocyte stages, 3T3-TRE-WT or 3T3-TRE-A212P cells were treated with 100 ng/ml of Dox. After 2 days of incubation, the cells were imaged under a fluorescence microscope (TS100-F with FL/Phase). Scale bar = 50 µm and applies to all panels.
(TIF)

Figure S7 **Dose-dependent rescue of adipogenic defect by Indomethacin in 3T3-A212P cells.** 3T3-CON and 3T3-A212P cells were grown to full confluency and subsequently subjected to standard DMI cocktail with indomethacin at the indicated concentrations. Indomethacin was included in the cells at the same concentrations until the indicated time points. Cells were then collected for Oil Red-O staining and extraction at the indicated time points. Data are presented as mean ± SEM. N = 2 independent experiments, each measured in triplicates. *p<0.05, **p<0.01, and ***p<0.001.
(TIF)

Table S1 **Complete list of up-regulated genes in 3T3-A212P cells.**
(XLS)

Table S2 **Complete list of down-regulated genes in 3T3-A212P cells.**
(XLS)

Table S3 **List of selected up-regulated genes related to inflammation response in 3T3-A212P cells.**
(DOC)

Table S4 **Down- or up-regulation of genes in target networks of PPARg, in 3T3-A212P cells.**
(XLS)

Table S5 **Down- or up-regulation of genes in target networks of TNF in 3T3-A212P cells.**
(XLS)

Table S6 **Down- or up-regulation of genes in target networks of IFNg in 3T3-A212P cells.**
(XLS)

Table S7 **Down- or up-regulation of genes in target networks of IL1b in 3T3-A212P cells.**
(XLS)
