# Supplementary figures and images for: Suppression of Adipogenesis by Pathogenic Seipin Mutant Is Associated with Inflammatory Response
Source: PLoS One. 2013 Mar 8;8(3):e57874. doi: 10.1371/journal.pone.0057874 (PMC3592919; doi:10.1371/journal.pone.0057874)

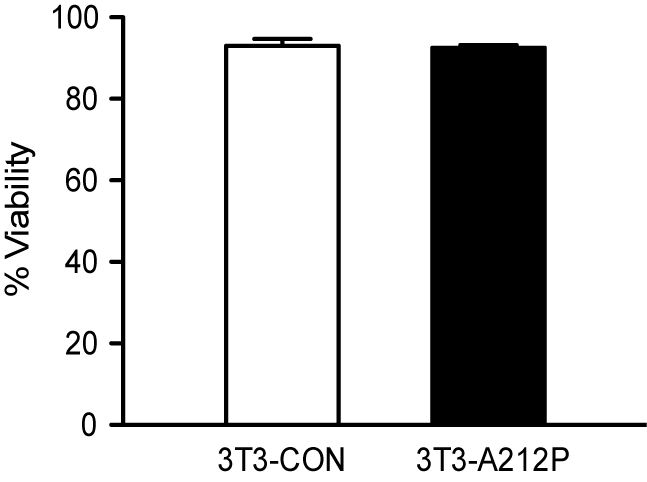

Supplement: Figure S1 — Normal cell viability in 3T3-A212P cells. 3T3-CON and 3T3-A212P cells were grown to full confluency and subsequently subjected to standard DMI cocktail. Fresh medium were added every 2 days and cells were trypsinized and collected at day 8. Cell count and cell viability were then performed using the Countess Automated Cell Counter according to the manufacturers instructions. Data are presented as mean ± SEM. N = 3 independent experiments, each measured in triplicates. (TIF) [file pone.0057874.s001.tif]

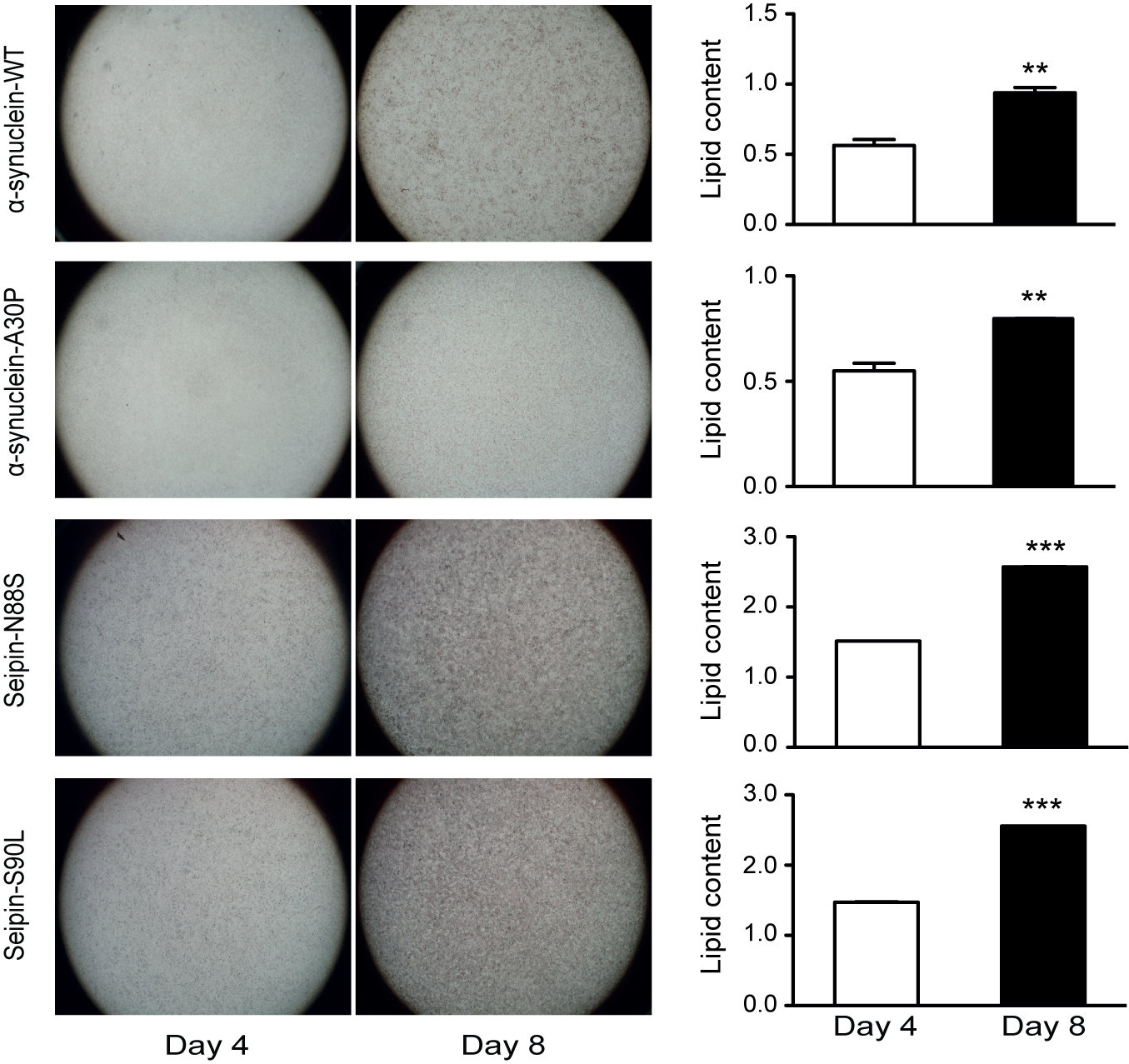

Supplement: Figure S2 — Overexpression of misfolding proteins does not inhibit adipogenesis. Stable 3T3-L1 cells expressing Seipin-N88S, Seipin-S90L, α-synuclein-WT or α-synuclein-A30P were grown to full confluency and subsequently subjected to standard DMI cocktail. Cells were then collected and subjected to Oil Red-O staining and extraction at the indicated time points. Data are presented as mean ± SEM. N = 2 independent experiments, each measured in duplicates. **p<0.01 and ***p<0.001. (TIF) [file pone.0057874.s002.tif]

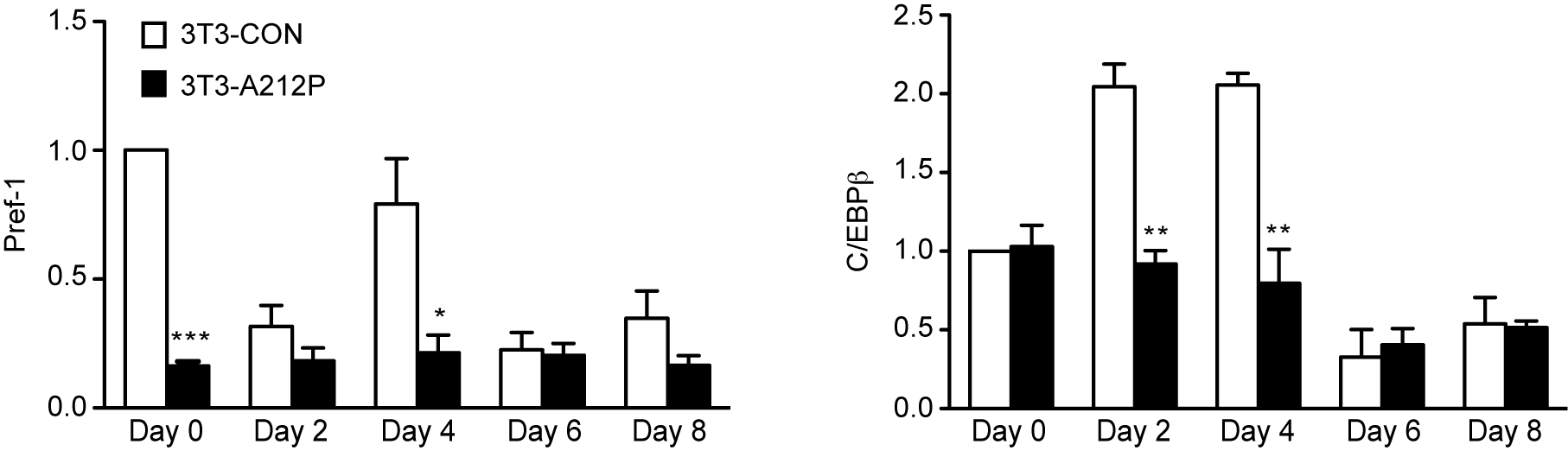

Supplement: Figure S3 — Early adipogenic markers are down-regulated in 3T3-A212P cells. Expression levels of Pref-1 and C/EBPβ in 3T3-CON and 3T3-A212P cells at the indicated time points during differentiation were assessed by real-time qPCR. Values were expressed as fold changes by normalizing to the level in control cells at Day 0. β-actin expression was used as an internal control. Data are presented as mean ± SEM. N = 3 independent experiments, each measured in triplicates. *p<0.05, **p<0.01, and ***p<0.001 vs. 3T3-CON cells at the same time points. (TIF) [file pone.0057874.s003.tif]

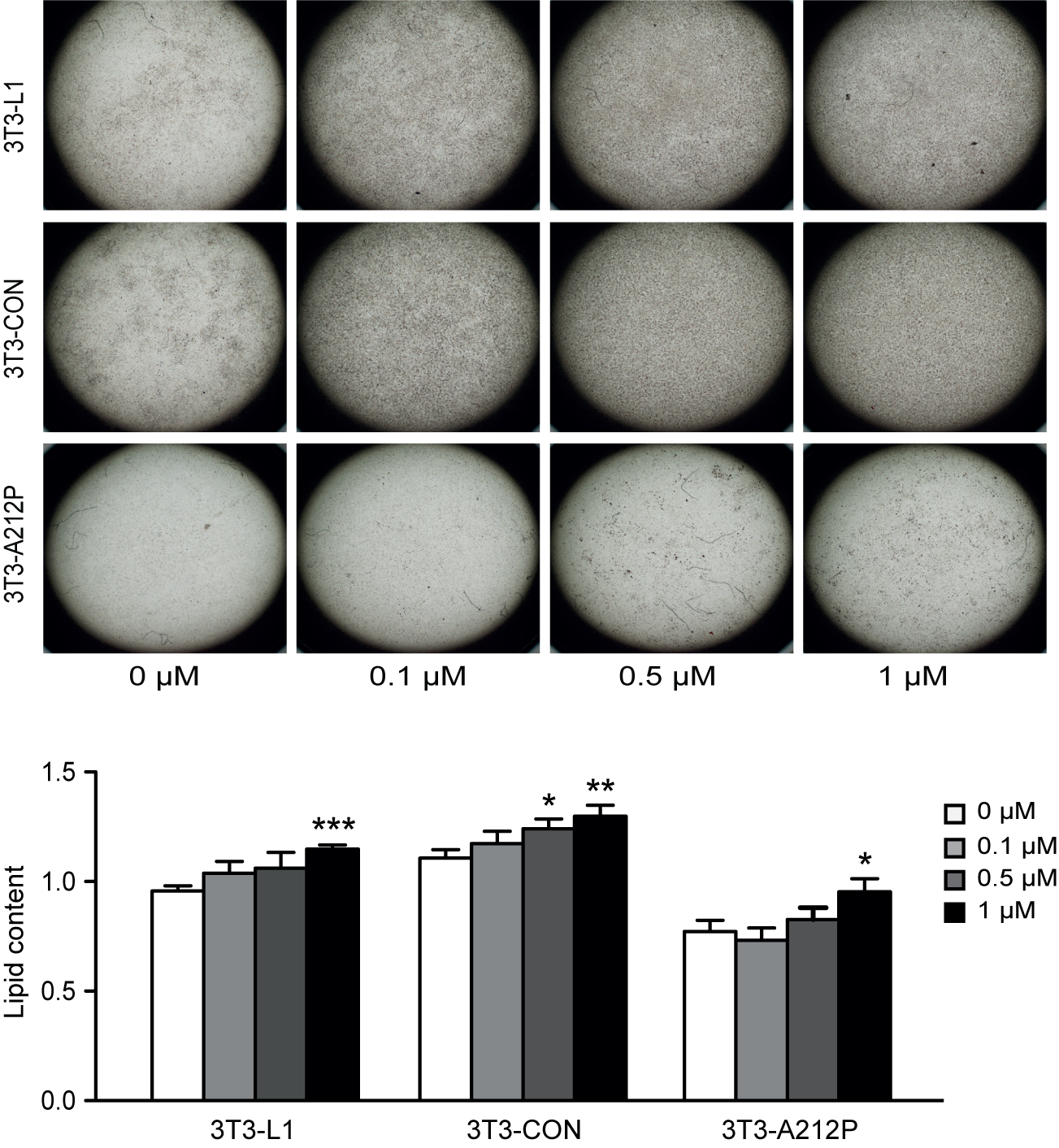

Supplement: Figure S4 — Dose-dependent rescue of adipogenic defect by pioglitazone in 3T3-A212P cells. 3T3-L1, 3T3-CON and 3T3-A212P cells were grown to full confluency and subsequently subjected to standard DMI cocktail with pioglitazone at the indicated concentrations. Pioglitazone was included throughout differentiation steps at the same concentrations. Cells were then collected for Oil Red-O staining and extraction at day 8. Data are presented as mean ± SEM. N = 3. *p<0.05, **p<0.01, and ***p<0.001. (TIF) [file pone.0057874.s004.tif]

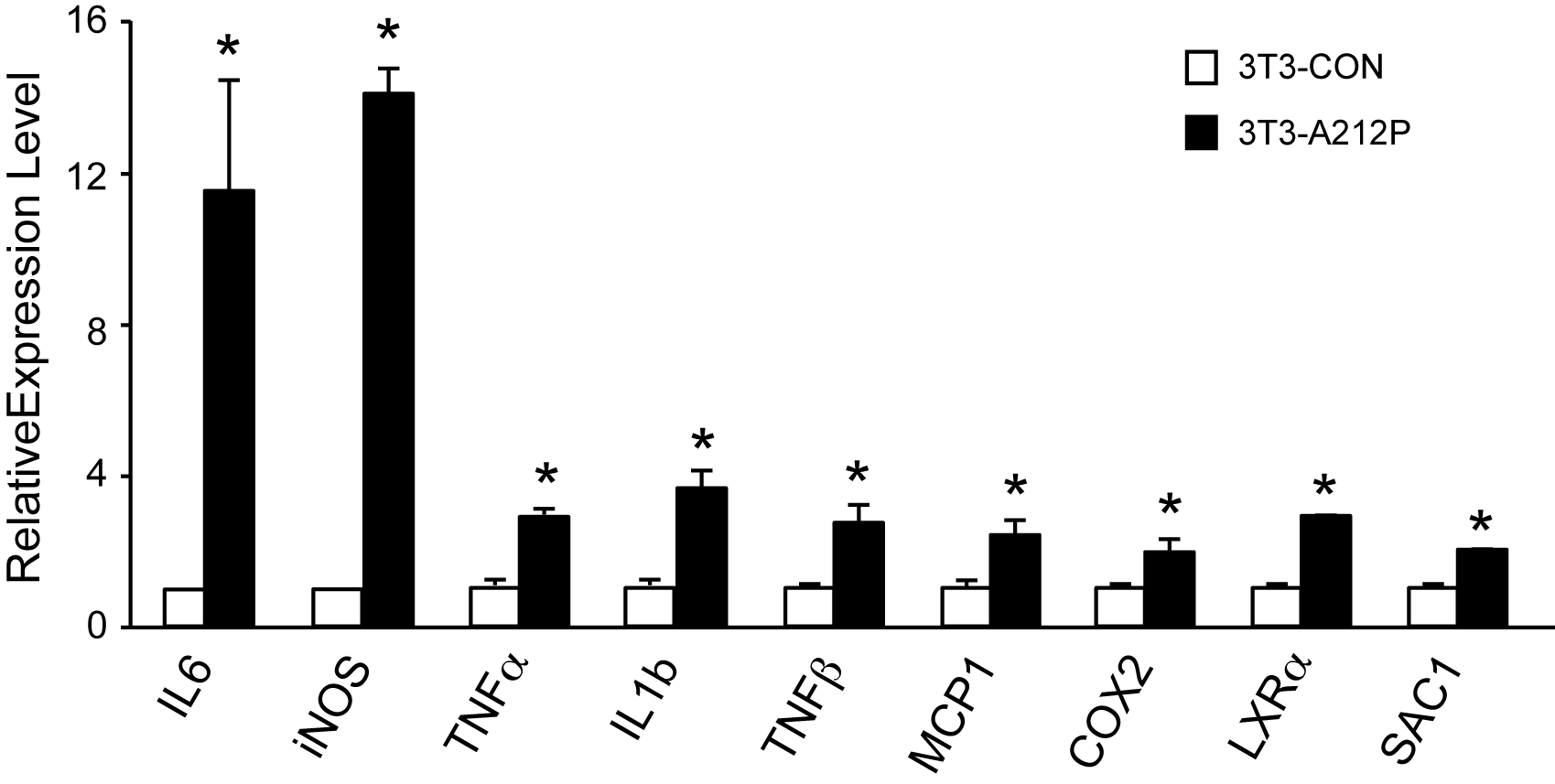

Supplement: Figure S5 — Seipin-A212P induces an inflammatory response in pre-adipocytes. At the pre-adipocyte stage, the total RNA of 3T3-CON and 3T3-A212P was extracted and expression of various inflammation response genes assessed by real-time qPCR. mRNA levels of different inflammation response markers were compared between 3T3-CON (white bar) and 3T3-A212P (black bar) cells. Data are presented as mean ± SD from three independent experiments. *p<0.05. (TIF) [file pone.0057874.s005.tif]

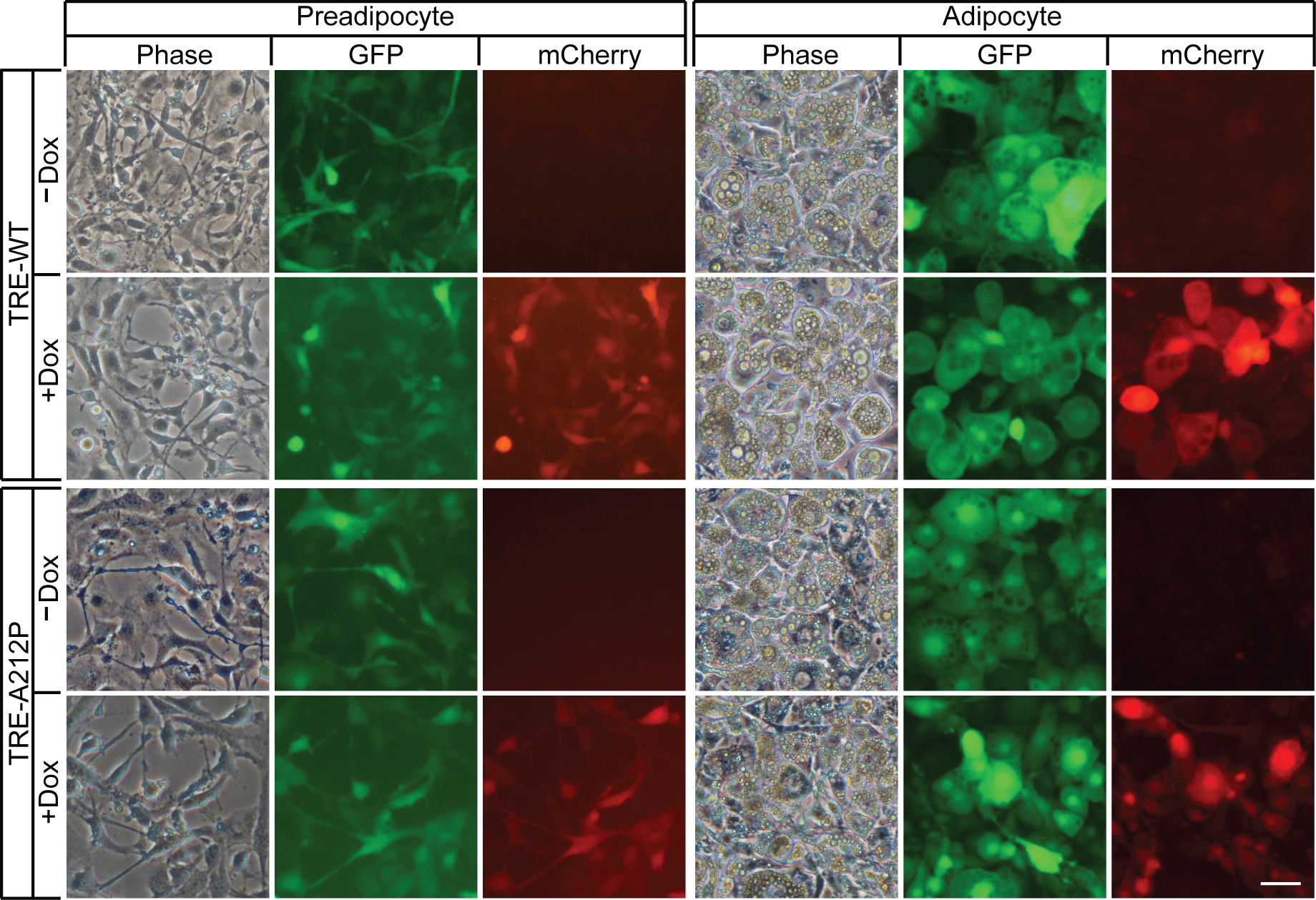

Supplement: Figure S6 — Induction of Seipin-WT and Seipin-A212P expression in the Tet-inducible stable cell lines. At the pre-adipocyte and mature adipocyte stages, 3T3-TRE-WT or 3T3-TRE-A212P cells were treated with 100 ng/ml of Dox. After 2 days of incubation, the cells were imaged under a fluorescence microscope (TS100-F with FL/Phase). Scale bar = 50 µm and applies to all panels. (TIF) [file pone.0057874.s006.tif]

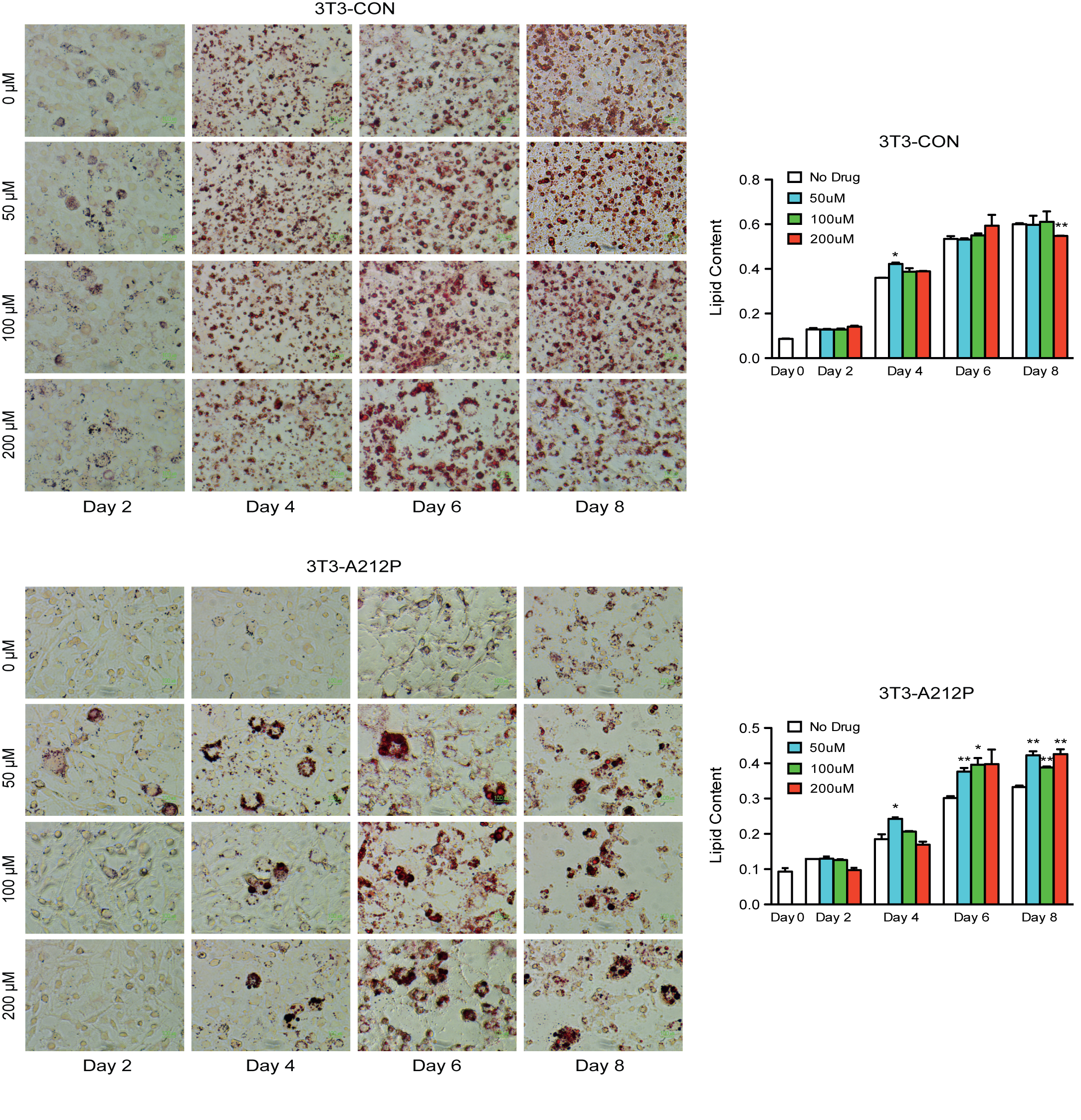

Supplement: Figure S7 — Dose-dependent rescue of adipogenic defect by Indomethacin in 3T3-A212P cells. 3T3-CON and 3T3-A212P cells were grown to full confluency and subsequently subjected to standard DMI cocktail with indomethacin at the indicated concentrations. Indomethacin was included in the cells at the same concentrations until the indicated time points. Cells were then collected for Oil Red-O staining and extraction at the indicated time points. Data are presented as mean ± SEM. N = 2 independent experiments, each measured in triplicates. *p<0.05, **p<0.01, and ***p<0.001. (TIF) [file pone.0057874.s007.tif]
