# Supplementary material for: Suppression of Adipogenesis by Pathogenic Seipin Mutant Is Associated with Inflammatory Response
Source: PLoS One. 2013 Mar 8;8(3):e57874. doi: 10.1371/journal.pone.0057874 (PMC3592919; doi:10.1371/journal.pone.0057874)
Supplement: Table S3 — List of selected up-regulated genes related to inflammation response in 3T3-A212P cells. (DOC) [file pone.0057874.s010.doc]

**Table S3. List of selected up-regulated genes related to inflammation response in 3T3-A212P cells**

| **Gene Symbol** | **Description** | **Changed Fold** |
| --- | --- | --- |
| Ehf | Ets homologous factor (Ehf), | 27 |
| Il1rl1 | Interleukin 1 receptor-like 1 (Il1rl1) | 20 |
| Il6 | Interleukin 6 (Il6) | 10 |
| Il1a | Interleukin 1 alpha (Il1a) | 10 |
| Il13ra2 | Interleukin 13 receptor, alpha 2 (Il13ra2) | 10 |
| Il33 | Interleukin 33 (Il33) | 9 |
| Tcfcp2l1 | Transcription factor CP2-like 1 (Tcfcp2l1) | 8 |
| Tnfsf18 | Tumor necrosis factor (ligand) superfamily | 8 |
| Tnfsf8 | Tumor necrosis factor (ligand) superfamily | 7 |
| Gata3 | GATA binding protein 3 (Gata3) | 4 |
| Runx1 | Runt related transcription factor 1 (Runx1) | 4 |
| Rnd1 | Rho family GTPase 1 (Rnd1) | 4 |
| Klf4 | Kruppel-like factor 4 (gut) (Klf4) | 4 |
| Notch3 | Notch gene homolog 3 (Drosophila) (Notch3) | 4 |
| Rgnef | Rho-guanine nucleotide exchange factor (Rgnef) | 3 |
| Gata2 | GATA binding protein 2 (Gata2) | 3 |
| Cebpd | CCAAT/enhancer binding protein (C/EBP), delta (Cebpd) | 3 |
| Junb | Jun-B oncogene (Junb) | 2 |
| Jun | Jun oncogene (Jun) | 2 |
